# Mediation Effect of Neutrophil Lymphocyte Ratio on Cardiometabolic Risk Factors and Cardiovascular Events

**DOI:** 10.1038/s41598-019-39004-9

**Published:** 2019-02-22

**Authors:** Teeranan Angkananard, Thunyarat Anothaisintawee, Atiporn Ingsathit, Mark McEvoy, Kongpop Silapat, John Attia, Piyamitr Sritara, Ammarin Thakkinstian

**Affiliations:** 1 Section for Clinical Epidemiology and Biostatistics, Faculty of Medicine, Ramathibodi Hospital, Mahidol University, Bangkok, Thailand; 2 Division of Cardiovascular Medicine, Faculty of Medicine, HRH Princess Maha Chakri Sirindhorn Medical Center, Srinakharinwirot University, Nakhon Nayok, Thailand; 3 Department of Family Medicine, Faculty of Medicine, Ramathibodi Hospital, Mahidol University, Bangkok, Thailand; 4 School of Medicine and Public Health, Faculty of Health and Medicine, University of Newcastle, Newcastle, New South Wales Australia; 5 Medical and Health Division, Electricity Generating Authority of Thailand, Nonthaburi, Thailand; 6 Hunter Medical Research Institute, University of Newcastle, New Lambton, New South Wales Australia; 7 Division of Cardiology, Department of Medicine, Faculty of Medicine, Ramathibodi Hospital, Mahidol University, Bangkok, Thailand

## Abstract

Neutrophil to lymphocyte ratio (NLR), an inflammatory biomarker, is associated with cardiovascular events (CVEs), but its causal pathway is unknown. We aimed to explore the extent to which NLR is directly associated with CVEs or mediated through diabetes mellitus (DM), hypertension (HT) and creatinine (Cr). The study used data on 2,501 subjects from the Electricity Generating Authority of Thailand cohort 2002–2012. Two causal pathways A: NLR→(DM→Cr→HT)→CVEs and B: NLR→(DM → HT→Cr)→CVEs were constructed. A generalized structural equation model and 1,000-replication bootstrapping were applied. The incidence rate of CVE was 8.8/1000/year. Prevalence rates of HT, DM, and chronic kidney disease were 45.1%, 23.6%, and 16.5%, respectively. The total effect of NLR on CVEs was explained partly (44%) by a direct effect and partly (56%) by an indirect effect through DM, HT and Cr. For pathway A, the direct OR of NLR on CVE was 1.25 (95% CI: 1.13, 1.39); the ORs for the indirect effects of NLR on CVEs mediated through DM, Cr, and poor-controlled HT were 1.06 (95% CI: 1.01, 1.11), 1.01 (95% CI: 1.00, 1.02), and 1.07 (95% CI: 1.01, 1.14) respectively. Results were similar for pathway B. Our findings demonstrate that roughly half of the relationship between NLR and CVEs may be mediated through DM, HT and Cr.

## Introduction

Cardiovascular diseases (CVDs) are the leading cause of mortality worldwide^1^. Approximately 17.7 million people died of CVD in 2015 representing 31% of all global deaths^1^ and costing about $316.1 billion/year^2^. Of these CVD deaths, an estimated 7.4, 6.7, 1.1, 0.4 and 2.1 million were due to coronary heart disease, stroke, hypertensive heart disease, cardiomyopathy and myocarditis and other CVDs, respectively^2^. Over three quarters of CVD deaths take place in low- and middle-income countries. Therefore, risk stratification and prognostication in CVD is important, so that individuals at high-risk can be accurately targeted for prevention.

The inflammatory response is a key mechanism in the pathogenesis of atherosclerosis and its progression^3^. Neutrophils secrete inflammatory mediators that can cause vascular wall degeneration. Conversely, lymphocytes have an anti-atherosclerotic role and thus regulate the inflammatory response^4^. The neutrophil to lymphocyte ratio (NLR), simply acquired from a complete blood count (CBC) can be an indicator of endothelial dysfunction^5^, and acute or chronic systemic inflammation^6^. It has recently emerged as an inflammatory biomarker and potential predictor of CVD risk in diverse populations, including people with acute coronary syndrome (ACS)^7^, stroke^8^, peripheral artery disease^9^, heart failure^10^, atrial fibrillation^11^, as well as those without CVD (e.g., cancers^6,12^, obstructive sleep apnea^13^). Previous studies indicate that an elevated NLR is associated with CVD outcomes^6,14^ (i.e. coronary artery disease (CAD), ACS, ischemic stroke, and composite CVEs) as well as significantly correlated with an increased risk of developing CVD risk factors^6^ (i.e. hypertension (HT)^15^, type 2 diabetes mellitus (DM)^16–18^, microalbuminuria^18,19^ and chronic kidney disease (CKD)^20^). Furthermore, it can reflect disease activity in patients with some chronic inflammatory disorders^6^ (e.g. Bechet disease^21^). Also, its values may be influenced by some medications which control HT^22^. In addition, a higher NLR could predict development of composite fatal and nonfatal CVEs in patients with stage 3–5 CKD^23^. We hypothesized that some of these associations with CVD might be due partly to an association between NLR and established CVD risk factors. None of the previous studies had assessed and quantified the extent to which the NLR effect on CVDs was mediated by CVD risk factors. This would allow us to understand more about the possible mechanisms (i.e., causal pathways) and estimate the contributions of multiple risk factors causing CVEs. Therefore, we conducted a study using data from a prospective cohort study to explore and quantify the direct effect of NLR on CVEs, and the indirect effect mediated through traditional CVD risk factors, i.e., HT, DM, and creatinine (Cr). Further potential pathways by which changes in CVD risk factors over time could mediate the effects of NLR on the risk of CVEs were investigated.

## Results

Baseline characteristics are described in Table 1. For baseline the Electricity Generating Authority of Thailand^24^ (EGAT 1/3), mean age was 59.1 ± 4.8 years, and the majority were males (75.1%). Baseline mean body mass index (BMI) and waist-hip ratio (WHR) were 24.4 ± 3.4 kg/m^2^ and 0.93 ± 0.06. Approximately a half of participants were smokers and alcohol drinkers and most of them exercised three times/week or more. Prevalences of HT, DM and dyslipidemia were 45.1%, 23.6%, and 84.4%, respectively. Mean serum Cr was 1.02 ± 0.43 mg/dL with 16.5% having CKD stage 3–5. A total of 219/2,501 subjects developed CVEs with an incidence rate of 8.8/1000/year. Mean NLR of those having CVEs was higher than those without CVEs (2.13 ± 0.12 vs. 1.79 ± 0.01, p-value = 0.04).

**Table 1:** Baseline characteristics of the studied participants of EGAT1 cohort.

Characteristics	EGAT1/3 n = 2,296	EGAT1/4 n = 1,901	EGAT1/5 n = 1,564
Age, years	59.1 ± 4.8	64.0 ± 4.7	68.8 ± 4.6
Sex, number (%)			
Male	1724 (75.1)	1404 (74.0)	1140 (72.9)
Female	572 (24.9)	497 (26.0)	424 (27.1)
Education number (%)			
≤High School	593 (25.8)	431 (22.7)	293 (18.7)
Vocational/Diploma	710 (30.9)	588 (30.9)	492 (31.5)
≥Bachelor	993 (43.2)	882 (46.4)	779 (49.8)
Income, number (%)			
Low income	478 (20.8)	631 (33.2)	809 (51.7)
Middle income	749 (32.6)	559 (29.4)	574 (36.7)
High income	1069 (46.6)	711 (37.4)	181 (11.6)
Marital status, number (%)			
Single	126 (5.5)	112 (5.9)	89 (5.4)
Married	1954 (85.1)	1562 (82.2)	1261 (80.6)
Widowed/ separate/divorce	216 (9.4)	227 (11.9)	218 (13.9)
BMI, kg/m^2^	24.4 ± 3.4	24.5 ± 3.5	23.9 ± 3.5
WHR	0.93 ± 0.06	0.94 ± 0.06	0.93 ± 0.06
SBP, mmHg	128.8 ± 18.9	134.4 ± 19.4	133.2 ± 18.3
DBP, mmHg	82.9 ± 11.4	80.7 ± 10.6	76.8 ± 10.3
Smoking, number (%)			
Non-smoker	1062 (46.3)	920 (48.4)	757 (48.4)
Ex-smoker	903 (39.3)	781 (41.1)	686 (43.9)
Current smoker	331 (14.4)	200 (10.5)	121 (7.7)
Alcohol, number (%)			
Non-drinker	1172 (51.1)	964 (50.7)	709 (45.3)
Ex-drinker	502 (21.9)	494 (25.9)	702 (44.9)
Current drinker	622 (27.1)	443 (23.3)	153 (9.8)
Exercise, number (%)			
None	447 (19.5)	154 (8.1)	448 (28.6)
1–2 times/week	412 (17.9)	287 (15.1)	187 (11.9)
≥3 times/week	1437 (62.6)	1460 (76.8)	929 (59.4)
Hypertension, number (%)			
No	1235 (54.9)	742 (40.1)	519 (33.5)
Poorly-controlled	721 (32.0)	707 (38.2)	514 (33.2)
Well-controlled	295 (13.1)	401 (21.7)	516 (33.3)
Diabetes Mellitus, number (%)			
No	1748 (76.4)	1414 (75.6)	1272 (81.4)
Yes	540 (23.6)	456 (24.4)	290 (18.6)
Dyslipidemia, number (%)			
No	359 (15.6)	459 (24.2)	313 (20.0)
Yes	1937 (84.4)	1442 (75.9)	1251 (79.9)
FPG, mg/dL	109.1 ± 35.1	102.2 ± 27.0	99.4 ± 22.6
TC, mg/dL	240.2 ± 43.5	211.0 ± 41.0	204.0 ± 43.2
TG, mg/dL	151.7 ± 106.1	135.1 ± 71.2	121.0 ± 61.3
LDL, mg/dL	156.8 ± 40.0	138.3 ± 37.9	131.7 ± 39.5
HDL, mg/dL	54.2 ± 14.7	57.8 ± 15.7	59.8 ± 16.6
NLR	1.6 ± 0.6	1.8 ± 0.7	2.0 ± 1.0
% Neutrophil	52.9 ± 8.0	61.6 ± 7.6	57.2 ± 8.7
% Lymphocyte	35.9 ± 7.3	35.8 ± 7.5	31.6 ± 8.0
Hb, g/dL	14.1 ± 1.8	13.6 ± 1.4	13.4 ± 1.5
Platelets, x10^3^ cells/mL	264.6 ± 72.3	225.8 ± 64.2	246.3 ± 61.9
Creatinine, mg/dL	1.02 ± 0.43	0.94 ± 0.37	1.02 ± 0.43
eGFR, mL/min / 1.73 m^2^	77.9 ± 18.2	80.9 ± 16.9	73.8 ± 15.6
CKD stage, number (%)			
Stage 1–2	1917 (83.5)	1678 (88.3)	1286 (82.2)
Stage 3–5	379 (16.5)	223 (11.7)	278 (17.8)
Uric acid, mg/dL	6.0 ± 1.3	6.2 ± 1.3	6.1 ± 1.5

Data are reported by mean ± SD or count (%); Low income, <20,000 Baht/month; Middle income, 20,000–49,999 Baht/month; High income, ≥ 50,000 Baht/month.

BMI, body mass index; WHR, waist hip ratio; SBP, systolic blood pressure; DBP, diastolic blood pressure; FPG, fasting plasma glucose; TC, total cholesterol; TG, triglyceride; LDL, low-density lipoprotein; HDL, high-density lipoprotein; NLR, neutrophil lymphocyte ratio; Hb, hemoglobin; eGFR, estimated glomerular filtration rate; CKD, chronic kidney disease.

### Multiple imputation

Data for 24 variables were missing, ranging from 8.7% to 65.5%. The multiple imputation by chained equations (MICEs) were initially constructed based on four completed variables (i.e., age, gender, history of taking nonsteroidal anti-inflammatory drugs (NSAIDs) and outcome data) with the missing at random assumption^25^ based on arbitrary missing-data patterns. Maximum fraction of missing information (FMI) and relative variance increase (RVI) values were 0.6105 and 1.5482 for both pathways (see Supplementary Table 1). Therefore 100 imputations were performed. A summary of imputed data compared with the initial incomplete data is illustrated in Supplementary Table 2.

## Pathway A

### CVEs outcome model

A univariate generalized structural equation model (GSEM) indicated 14/19 co-variables significantly associated, with CVEs but only three variables (i.e., age, smoking, and history of taking NSAIDs) were finally kept in the multivariate GSEM that already contained NLR, Cr, HT and DM, see Supplementary Table 3.

### Mediation model

Univariate and multivariate versions of GSEM were performed for three mediators, i.e., DM, Cr, and HT (well- and poorly-controlled HT vs. non-HT). For univariate GSEM analysis, 14, 14, and 17 co-variables were significantly associated with DM, Cr and HT, respectively (data not shown). However, only five, four, and eleven co-variables were considered significant in the multivariate GSEM of DM, Cr, HT mediators, respectively which already included NLR, see Supplementary Table 4.

Direct effect and the average causal mediation effects (ACMEs) were estimated (see Table 2 and Supplementary Fig. 1A) indicating a significant direct effect of NLR on CVEs with an OR of 1.25 (95% CI: 1.13, 1.39). For the ACMEs with one mediator, the coefficients of NLR and CVEs through DM, Cr, and poorly-controlled HT were respectively 0.056 (95% CI: 0.020, 0.121), 0.014 (95% CI: 0.006, 0.022) and 0.068 (95% CI: 0.023, 0.148) yielding corresponding ORs of 1.058 (95% CI: 1.006, 1.110), 1.014 (95% CI: 1.006, 1.022), and 1.071 (95% CI: 1.005, 1.136). This indicates that each unit increase in NLR would increase risk of CVEs through DM, Cr, and poorly-controlled HT by around 6%, 1% and 7%, respectively, and would directly increase risk of CVEs by about 25%.

**Table 2:** Results of mediation analysis: direct, indirect and total effects of NLR on CVEs mediated by diabetes mellitus, creatinine, and hypertension via pathway A.

Path	Effect	SE	z	P-value	Bootstrapping	Odds Ratio	95% CI	Proportion mediated (%)
					Bias Corrected 95%CI			
Direct effect	0.2246	0.0535	4.20	<0.001	0.1235, 0.3306	1.2518	1.1315, 1.3918	44.73
Indirect effects								
Overall	0.2775	0.1344	2.06	0.039	0.0505, 0.5864	1.3198	1.0141, 1.7176	55.27
Through DM	0.1030	0.0402	2.56	0.010	0.0423, 0.2127	1.1085	1.0199, 1.1971	20.51
Through Cr	0.0248	0.0083	3.00	0.003	0.0111, 0.0443	1.0251	1.0084, 1.0418	4.94
Through HT_1_	0.0400	0.0759	0.53	0.598	−0.1144, 0.1842	1.0409	0.8849, 1.1968	7.97
Through HT_2_	0.1097	0.0427	2.56	0.010	0.0438, 0.2122	1.1159	1.0209, 1.2108	21.85
One mediator model								
NLR→DM→CVEs	0.0564	0.0250	2.25	0.024	0.0203, 0.1209	1.0580	1.0058, 1.1102	11.23
NLR→ Cr →CVEs	0.0138	0.0041	3.37	0.001	0.0060, 0.0219	1.0139	1.0058, 1.0220	2.75
NLR→HT_1_→CVEs	0.0315	0.0605	0.52	0.602	−0.0886, 0.1478	1.0320	0.9089, 1.1551	6.27
NLR→ HT_2_→CVEs	0.0681	0.0306	2.23	0.026	0.0227, 0.1477	1.0705	1.0054, 1.1355	13.56
Two mediators model								
NLR→DM→Cr → CVEs	0.0031	0.0014	2.19	0.028	0.0012, 0.0069	1.0031	1.0003, 1.0058	0.62
NLR→DM→HT_1_→CVEs	0.0072	0.0142	0.51	0.612	−0.0180, 0.0397	1.0072	0.9791, 1.0354	1.43
NLR→DM→HT_2_→CVEs	0.0349	0.0161	2.16	0.031	0.0129, 0.0791	1.0355	1.0025, 1.0685	6.95
NLR→Cr→HT_1_→CVEs	0.0011	0.0023	0.45	0.649	−0.0027, 0.0076	1.0011	0.9965, 1.0056	0.22
NLR→Cr→HT_2_ →CVEs	0.0054	0.0033	1.66	0.097	0.0015, 0.0151	1.0054	0.9989, 1.0119	1.08
Three mediators model								
NLR→DM→Cr→HT_1_ →CVEs	0.0002	0.0005	0.46	0.646	−0.0006, 0.0016	1.0002	0.9992, 1.0012	0.04
NLR→DM→Cr→HT_2_ →CVEs	0.0012	0.0007	1.64	0.101	0.0004, 0.0035	1.0012	0.9998, 1.0027	0.24
Total effect	0.5021	0.1388	3.62	<0.001	0.2480, 0.7941	1.6522	0.2301, 0.7741	100

CI, confidence interval; NLR, neutrophil-lymphocyte ratio; CVEs, cardiovascular events; DM, diabetes mellitus; Cr, creatinine; HT1, well-controlled hypertension; HT2, poorly-controlled hypertension.

The pathway with two, but not three mediators also showed significant effects of NLR on CVEs through DM-Cr (NLR→DM →Cr→CVEs) and DM-poorly-controlled HT (NLR→DM→poorly-controlled HT→CVEs) with the ACMEs of 0.003 (95% CI: 0.001, 0.007) and 0.035 (95% CI: 0.013, 0.079), respectively. The corresponding ORs were 1.003 (95% CI: 1.000, 1.006) and 1.036 (95% CI: 1.003, 1.069), implying that each unit increase in NLR level would increase risk of CVEs through DM-Cr, and DM-poorly-controlled HT by around 0.3% and 4%, respectively. However, the models of NLR→Cr→well-controlled HT→CVEs and NLR→Cr→poorly-controlled HT→CVEs were not significant.

Considering the total effect of NLR on CVEs, the direct effect contributed about 44.73% whereas the remainder of the effect was mediated through poorly-controlled HT, DM, well-controlled HT and Cr contributing substantial effects about 21.85%, 20.51%, 7.97% and 4.94%, respectively. Overall, the ORs and their 95% CIs of total ACMEs or total indirect effects of NLR on CVEs adjusting for DM, HT and Cr was 1.32 (95% CI: 1.01, 1.72), see Table 2.

## Pathway B

This pathway showed similar effects of NLR on CVEs as pathway A except for three mediators which indicated that the effects of NLR on CVEs through DM, HT, and Cr mediators (NLR→DM →well- or poorly-controlled HT→Cr→CVEs) were significant with ACMEs of 0.001 (95% CI: 0.000, 0.003) and 0.002 (95% CI: 0.001, 0.003), see Table 3 and Supplementary Fig. 1B. These corresponded to ORs of 1.001 (95% CI: 1.000, 1.003) and 1.002 (95% CI: 1.001, 1.003), respectively. As before, this indicates that each unit increase in NLR intensifies risk of CVEs through DM-well-controlled HT-Cr and DM-poorly-controlled HT-Cr by around 0.1% and 0.2%.

**Table 3:** Results of mediation analysis: direct, indirect and total effects of NLR on CVEs mediated by diabetes mellitus, creatinine, and hypertension via pathway B.

Path	Effect	SE	z	P-value	Bootstrapping	Odds Ratio	95% CI	Proportion mediated (%)
					Bias Corrected 95% CI			
Direct effect	0.2246	0.1298	4.20	<0.001	0.1235, 0.3306	1.2518	1.1315, 1.3918	43.71
Indirect effects								
Overall	0.2892	0.1344	2.21	0.027	0.0759, 0.5896	1.3318	1.0327, 1.7175	56.29
Through DM	0.1052	0.0407	2.58	0.010	0.0434, 0.2159	1.1109	1.0211, 1.2008	20.47
Through Cr	0.0264	0.0065	4.06	<0.0001	0.0146, 0.0399	1.0267	1.0147, 1.0408	5.15
Through HT_1_	0.0457	0.0005	0.61	0.541	−0.1050, 0.1875	1.0468	0.8921, 1.2015	8.90
Through HT_2_	0.1119	0.0020	2.56	0.010	0.0458, 0.2145	1.1184	1.0236, 1.2131	21.77
One mediator model								
NLR→ DM→ CVEs	0.0564	0.0250	2.25	0.024	0.0203, 0.1209	1.0580	1.0205, 1.1285	10.98
NLR→ Cr → CVEs	0.0131	0.0040	3.25	0.001	0.0052, 0.0212	1.0131	1.0052, 1.0214	2.55
NLR→ HT_1_→CVEs	0.0321	0.0615	0.52	0.602	−0.0919, 0.1513	1.0326	0.9122, 1.1633	6.25
NLR→ HT_2_→CVEs	0.0714	0.0314	2.23	0.026	0.0243, 0.1502	1.0739	1.0246, 1.1621	13.89
Two mediators model								
NLR→DM→Cr →CVEs	0.0026	0.0013	2.07	0.039	0.0009, 0.0062	1.0026	1.0009, 1.0062	0.51
NLR→DM→HT_1_→CVEs	0.0074	0.0146	0.51	0.612	−0.0183, 0.0401	1.0075	0.9819, 1.0409	1.44
NLR→DM→HT_2_→CVEs	0.0360	0.0166	2.17	0.030	0.0135, 0.0818	1.0367	1.0136, 1.0853	7.01
NLR→HT_1_→Cr →CVEs	0.0051	0.0018	2.80	0.005	0.0019, 0.0089	1.0051	1.0019, 1.0089	0.99
NLR →HT_2_→Cr →CVEs	0.0029	0.0012	2.43	0.015	0.0011, 0.0056	1.0029	1.0011, 1.0056	0.56
Three mediators model								
NLR→DM→HT_1_→Cr →CVEs	0.0012	0.0006	2.08	0.038	0.0004, 0.0027	1.0012	1.0004, 1.0027	0.23
NLR→DM→HT_2_→Cr →CVEs	0.0015	0.0007	2.22	0.027	0.0006, 0.0032	1.0015	1.0006, 1.0032	0.29
Total effect	0.5138	0.1343	3.81	<0.001	0.2669, 0.7872	1.6671	1.2813, 2.1692	100

CI, confidence interval; NLR, neutrophil-lymphocyte ratio; CVEs, cardiovascular events; DM, diabetes mellitus; Cr, creatinine; HT1, well-controlled hypertension; HT2, poorly-controlled hypertension.

The direct effect of NLR on CVEs contributed about 43.71%, whereas the remaining effect was mediated through poorly-controlled HT, DM, well-controlled HT and Cr contributing substantial effects about 21.77%, 20.47%, 8.90% and 5.15%, respectively. Overall, the ORs and their 95% CIs of total indirect effects of NLR on CVEs through DM, HT and Cr mediators was 1.33 (95% CI: 1.03,1.72), see Table 3.

## Discussion

Our findings provide the first quantification of possible causal associations between NLR and CVEs, through known modifiable cardiometabolic risk factors. Multiple mediators were considered using parallel and serial mediator models for causal effects of NLR on CVEs. Direct and indirect effects mediated through poorly-controlled HT, DM and serum Cr were estimated.

Several previous studies have observed that elevated NLR is associated with higher risk of CVEs^23,26^ as well as greater risk of insulin resistance in DM^27–30^, CKD^20^, and HT^31,32^. Collectively, our results support the idea that inflammation and oxidative stress play a vital role in the pathophysiology of how DM^33,34^, CKD^35^, HT^31^, and endothelial dysfunction influence the pathogenesis and progression of atherosclerosis and CVDs^36,37^.

This mediation analysis used data from a prospective cohort study (EGAT1). The EGAT1 cohort gathers detailed longitudinal information every 5 years. NLR was the independent variable, DM, Cr and HT were mediators, and CVEs was the outcome of interest. All variables were in sequence; i.e., NLR and all risk factors were derived from the early phase of the cohort and CVEs developed thereafter. The measures of all mediators and CVE outcomes were performed and tested for the reciprocity of mediating effects at all surveys. In addition, two causal pathways were constructed in our mediation analysis and the overall results looked similar. The temporality assumption in our study was therefore robust^38,39^.

From prior evidence, the relationship between inflammatory biomarkers and later CVEs has been attenuated partly, but not completely by classical CVD risk factors^40,41^. However, no studies to date had investigated and quantified the extent to which the associations are mediated by these risk factors. The 2 pathways tested here are consistent in showing:

- HT, DM and renal function all impact CVEs with approximately equal weighting.
- The NLR most strongly impacts HT, then DM, and only minimally impacts renal function.
- Combining these indicates that about 45% of the effect of NLR is directly on CVE, or potentially through mechanisms other than those described here; the remainder of the effect is roughly equally mediated through DM and HT, and only a small proportion through renal function.

CKD and HT have a cyclic cause and effect relationship. Both high-normal BP [systolic blood pressure (SBP) 130–139 mmHg or diastolic blood pressure (DBP) 85–89 mmHg] and HT (SBP ≥ 140 or DBP ≥ 90 mmHg) are independent risk factors for worsening renal function as evidenced by a direct relationship with BP severity^42^. Long standing poorly-controlled HT leads to high intra-glomerular pressure and impaired glomerular filtration^43^. However, renal function decline can also worsen BP control due to volume expansion and increased systemic vascular resistance^44^. This emphasizes the importance of well-controlled BP for prevention of future CVEs. It is interesting that in our study the causal relationship between renal function and HT was much stronger than in the reverse direction.

This study has many strengths. This was a prospective cohort study which allowed us to follow up any changes in risk factors and define the correct temporal association with the outcome. This is the first analysis of more than two mediators using serial mediator models for causal sequences of CVEs. These models allow us to understand more about the relative contributions of multiple risk factors causing CVEs in a single complex model. They also allow us to test the causal directions of one mediator on the others. Moreover, the biomarker used here is inexpensive (i.e., NLR) and easily obtained from a routine laboratory test. Likewise in the previous study^45^ of healthy persons without dyslipidemia, but with elevated high-sensitivity C-reactive protein levels, lowering these levels with statin could significantly reduce the incidence of CVEs. Our results also raise the possibility that tailoring interventions aimed at reducing inflammation (lowering NLR) and slowing the decline in renal function in patients with or without DM and HT may retard CVD progression.

There are also some potential limitations. The study participants of the EGAT1 cohort were not representative of all Thai people, hence, the generalizability is uncertain. Missing CBC data in the first and second survey may have caused some bias in estimations and the sequence of NLR and mediators might not be exactly sequential. However, CVEs outcome occurred subsequently to ascertainment of NLR and mediators. The ideal period to estimate and apply NLR for predicting CVEs should be closer to those events^9,46^, so the temporal relationship of NLR or those mediators with CVEs outcome could be defended. With our limited data, we could not test mediation effects of other possible mediators, such as genetic factors (e.g. Duffy antigen variant locus^46^) or cancer history, which has common basic molecular pathways with CVD^47^ through chronic inflammatory process. It is possible that a significant direct NLR effect is mediated through other known or unknown risk factors which remains to be determined and which were not included in our causal schema. For example, baseline microalbuminuria (MAU) was independently associated with lower eGFR and future CVEs^48–50^, but was not included as one mediator in our analysis. In our cohort, urine dipstick was collected as a qualitative measurement of proteinuria instead of MAU. Further mediation study of MAU may provide the better causal pathways and prediction. Moreover, we used an office-based BP measurement in the baseline EGAT protocol which may have caused us to misdiagnose some hypertensive patients with masked uncontrolled HT as well-controlled HT. Additionally, the new clinical HT guideline^51^ has changed the threshold to SBP ≥ 130 mm Hg or DBP ≥ 80 mmHg. However, this study used the previous definition of HT for classifying patients^52^.

## Conclusion

Our study demonstrates a direct relationship between the NLR and CVEs, as well as indirectly through DM, HT and Cr. However, nearly half of the NLR effect operates independently from the 3 established cardiometabolic risk factors modelled here, raising the possibility that healthy individuals with elevated NLR levels are at risk for CVEs and should receive primary prevention. Interestingly, the mediated effect of NLR through well-controlled HT was noted in patients with worsening renal function. Based on our limited data, we strongly recommend exploring this relationship with other possible mediators and confirm this causal relationship in other datasets.

## Methods

### Study design and setting

Data from a 16-year-prospective study of employees at the Electricity Generating Authority of Thailand (EGAT)^24^ was used, which comprised of three successive cohorts; EGAT1, EGAT2 and EGAT3 starting in 1985, 1998 and 2009, respectively. The primary aim of these cohorts was to study risk factors and incidence of cardiovascular disease. The EGAT cohorts were located at the headquarters of EGAT in the Bangkok metropolitan area and at three different sites in Western and Northern Thailand. In this study, we focused on the data from EGAT1, which had follow-up CBC data collected from 2002–2012. The EGAT1 cohort was resurveyed every 5 years, i.e., in 1997(EGAT1/2), 2002(EGAT1/3), 2007(EGAT1/4) and 2012(EGAT1/5). Participants were excluded if they had CVDs at baseline, including CAD, myocardial infarction (MI), receiving revascularization, or any types of stroke, prior history of local or systemic infection within 3 months, and history of hematologic disorder or solid tumor. As a result, 3,025 out of 7,824 participants were eligible for this study. We excluded 524 participants enrolled in our baseline cohort (EGAT 1/3) whose NLR data were not available or had CVEs at/before, leaving totally 2,501 participants. Of these 2,296, 1,901 and 1,564 participants participated with the EGAT1/3, 1/4 and 1/5, respectively.

All participants completed a self-administered questionnaire which consisted of demographic data, risk and preventive behaviors for CVD, family medical history, underlying disease and medication history. Participants also underwent a physical examination which included blood pressure (BP), heart rate, weight, height and waist and hip circumference, electrocardiograms, chest X-rays; and provided a fasting (12-hour overnight) blood sample which measured CBC, neutrophil and lymphocyte count, plasma glucose, lipid profile, serum creatinine, estimated glomerular filtration rate (eGFR)^53^, uric acid, and a dipstick urinalysis for urinary protein.

#### Outcomes of interest

The primary outcome of interest was CVEs which included fatal/non-fatal MI, other coronary events (coronary artery bypass graft, percutaneous coronary interventions, angina necessitating emergency room visits), or any fatal/non-fatal cerebrovascular event. Cause of death data was adjudicated by consensus of a blinded panel.

#### Study Factor

The NLR was our primary factor of interest, calculated by a ratio of total number or percent count of neutrophils to total number or percent count of lymphocytes, obtained from the CBC. All blood samples were collected in ethylenediaminetetraacetic acid tubes and processed using an automated machine (Technicon H-1) within 6–8 h after blood collection. The coefficients of variation was 1.61^54,55^.

#### CVD risk factors

DM^56^ was diagnosed as fasting blood sugar ≥126 mg/dl (7.0 mmol/L), or hemoglobin A1C ≥ 6.5% (48 mmol/mol), or taking antidiabetic drugs during the past 2 weeks.

HT^57^ was diagnosed if SBP ≥ 140 mmHg or DBP ≥ 90 mmHg, or taking antihypertensive medications. BP was measured following standard recommendation using automated BP machines (Omron®) which were calibrated before the physical examination. Participants were classified as normotensive (SBP < 140 mmHg and DBP < 90 mmHg without antihypertensive medication), poorly-controlled HT (SBP ≥ 140 mmHg and/or DBP ≥ 90 mmHg and on antihypertensive medication); or well-controlled HT (SBP < 140 mmHg and DBP < 90 mmHg with antihypertensive medication).

Dyslipidemia was classified if they had at least one of four of the following criteria^58^: (1) High-density lipoprotein cholesterol (HDL-C) < 50 mg/dL in female or HDL-C < 40 mg/dL in male; and/or (2) Low-density lipoprotein cholesterol ≥160 mg/dL; and/or (3) Triglyceride ≥150 mg/dL; and/or 4) used any lipid-lowering medications.

CKD^59^ was defined and categorized according to eGFR. The eGFR was calculated using the Chronic Kidney Disease Epidemiology Collaboration (CKD-EPI: 2009) equation.

Anemia^60^ was defined as hemoglobin level <12.0 g/dL in non-pregnant women and <13.0 g/dL in men.

Age in years was calculated by subtracting survey date from date of birth. BMI was calculated as weight/height (m^2^). Underweight, normal weight, overweight and obesity were diagnosed when BMI < 18.5, 18.5–22.9, 23–24.9 and ≥25 kg/m^2^, respectively^61^. Waist-hip ratio (WHR)^62^ was calculated from the recorded waist circumference divided by hip circumference in centimeters. Smoking was categorized as smoker (i.e., current plus ex-smoker) and non-smoker, obtained by self-report. Along with alcohol drinker, defined as ≥5 alcoholic drinks for males or ≥4 alcoholic drinks for females within a couple of hours of each other on at least 1 day in the past month and was classified similar to smoking status^63^. Physical activity was grouped according to self-reported frequency of exercise per week as ≤1–2 times/week and ≥3 times/week.

#### Imputation of data

The forward and backward carry over methods were applied to complete missing data as much as possible, see Supplementary Table 5. Twenty-four variables remained missing and were imputed by a simulation-based approach with MICE method^64^ for longitudinal data^65^ with the missing at random assumption^25^ (see Supplementary Table 6 and Appendix A). Performance of imputation was measured by the largest FMI coefficient and the average RVI which quantifies the uncertainty of the values estimated from multiple imputations. All imputations were performed using MICE commands in STATA^66^.

### Statistical analysis

The baseline demographic data was analyzed and reported as mean ± SD or median and interquartile range where appropriate for continuous data and frequency with percentage was used for categorical data. The incidence rate and the cumulative incidence of having CVEs were calculated.

#### Mediation analysis

**Figure 1:**
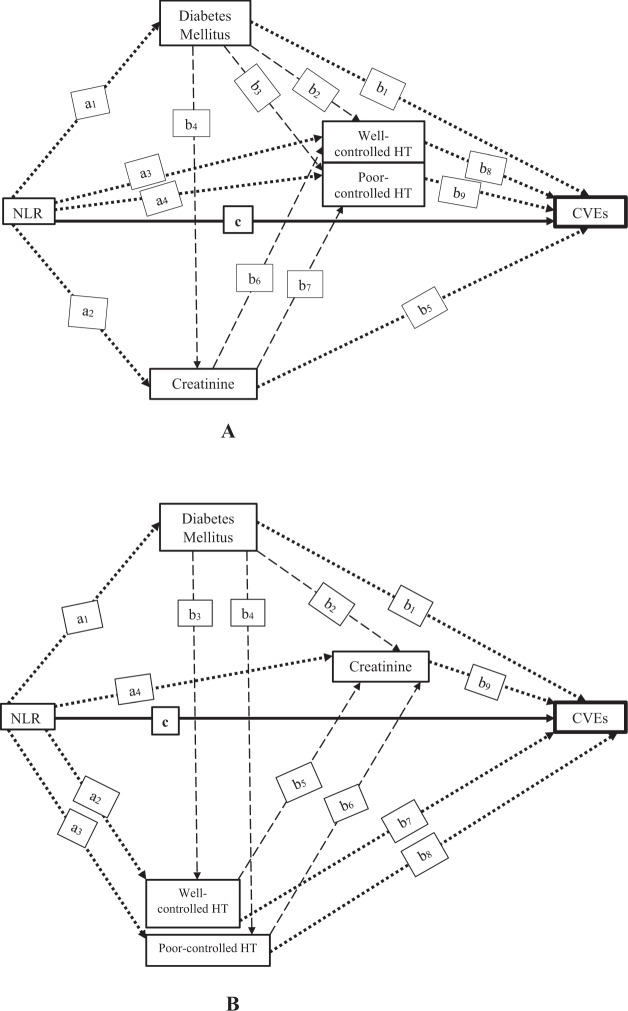
Causal pathway diagrams of NLR and CVEs. (**A**) Pathway A: NLR → (DM → Cr → HT) → CVEs. (**B**) Pathway B: NLR →(DM → HT→ Cr) → CVEs. The a_1_–a_4_, b_1_–b_9_ and c depict as path coefficients; NLR, neutrophil-lymphocyte ratio; CVEs, cardiovascular events. Cardiometabolic risk factors of CVEs (diabetes mellitus, creatinine, well- and poorly-controlled HT) are mediators on the causal pathway between exposure (NLR) and CVEs (outcome) with measured confounders, including age, gender, education, income, physical activity, smoking and alcohol consumption, history of taking NSAIDs, BMI, WHR, dyslipidemia, anemia, platelet count and uric acid level. The direct effect is illustrated by the solid arrow, and indirect effects using parallel multiple mediator models are shown by dotted arrows and serial multiple mediator models are represented as dashed arrows.

A multiple mediation analysis^67,68^ was applied to explore the causal pathways as described in Fig. 1A,B and Supplementary Fig. 1. Two causal pathways were constructed, i.e., pathway A: NLR→(DM → Cr→ HT)→CVEs (Fig. 1A) and pathway B: NLR→(DM → HT→Cr)→ CVEs (Fig. 1B). For both pathways, NLR was the independent variable, DM, Cr and HT were multiple mediators, and CVE was the outcome of interest. The outcome (CVEs) and mediation (DM, HT, Cr) models were constructed using GSEM. The set of confounders considered one-by-one in the univariate analysis of GSEM were age, gender, education, income, physical activity, smoking and alcohol consumption, history of taking NSAIDs, BMI, WHR, dyslipidemia, anemia, platelet count, and uric acid level. Variables having P-value < 0.1 were simultaneously considered in multivariate GSEM. Forward stepwise selection was applied to select and keep only significant variables in each mediation and outcome model.

For pathway A, the NLR was directly and parallelly fitted on DM and HT mediators using a GSEM with logit link, and Cr with identity link. Then, DM was serially fitted on Cr and both were next serially fitted on HT. Finally, all these variables were fitted on CVE using a logit link. Equations for pathway B were constructed similarly as for pathway A, except that DM was fitted on HT, and both were then fitted on Cr. Co-variables including age, gender, education, income, physical activity, smoking and alcohol consumption, history of taking NSAIDs, BMI, WHR, dyslipidemia, anemia, platelet count and uric acid were considered for inclusion in these serial multiple mediator models (SMMM) if they were significantly associated with at least one mediator or outcome. Serial coefficients were estimated accordingly and resulted in 11 causal pathways, see Supplementary Tables 7, 8 and Supplementary Fig. 2A,B.

These estimated coefficients were then used to estimate direct effect and ACMEs of NLR on CVEs using the product of coefficients method^69^. A bootstrap analysis was applied to estimate ACMEs across 1000 replications, and the corresponding 95% confidence interval (CI) was determined using a bias-corrected bootstrap technique^70^. All analyses were performed using STATA version 15.0. A P value < 0.05 was considered statistically significant. STATA codes for mediation analysis and bootstrapping were illustrated in Appendix B.

The study was approved by the Institutional Review Board of Ramathibodi’s Ethical Committee, Mahidol University, Thailand on January 11, 2017 and implemented in accordance with the ethical standards of the 1964 Declaration of Helsinki and its later amendments. All participations in the study were absolutely voluntary and gave their written informed consents including allowance to use their reserved blood samples for future additional testing.

## Supplementary information

SUPPLEMENTARY MATERIAL CONTENTS

## Data Availability

This prospective study used the demographic, medical and laboratory data of participants from EGAT projects which belong to the Faculty of Medicine, Ramathibodi Hospital, Mahidol University and restricted by the Ramathibodi’s Ethical Committee in order to protect patient privacy. Data are available from the Head of EGAT projects (Prof. Piyamitr Sritara) for researchers who meet the criteria for access to confidential data.
