# Supplementary material for: Mediation Effect of Neutrophil Lymphocyte Ratio on Cardiometabolic Risk Factors and Cardiovascular Events
Source: Sci Rep. 2019 Feb 22;9:2618. doi: 10.1038/s41598-019-39004-9 (PMC6384908; doi:10.1038/s41598-019-39004-9)
Supplement: Supplementary file 1 — SUPPLEMENTARY MATERIAL CONTENTS [file 41598_2019_39004_MOESM1_ESM.pdf]

SUPPLEMENTARY MATERIAL CONTENTS

Title: Mediation Effect of Neutrophil Lymphocyte Ratio on Cardiometabolic Risk Factors and Cardiovascular Events

Author list:

Teeranan Angkananard, MD, PhD [1,2]

Thunyarat Anothaisintawee, MD, PhD [1,3*]

Atiporn Ingsathit, MD, PhD [1]

Mark McEvoy, PhD [4]

Kongpop Silapat, MD [5]

John Attia, MD, PhD [4,6]

Piyamitr Sritara[7]

Ammarin Thakkinstian, PhD [1]

[1] Section for Clinical Epidemiology and Biostatistics, Faculty of Medicine, Ramathibodi Hospital, Mahidol University, Bangkok, Thailand

[2] Division of Cardiovascular Medicine, Faculty of Medicine, HRH Princess Maha Chakri Sirindhorn Medical Center, Srinakharinwirot University, Nakhon Nayok, Thailand

[3] Department of Family Medicine, Faculty of Medicine, Ramathibodi Hospital, Mahidol University, Bangkok, Thailand

[4] Center for Clinical Epidemiology and Biostatistics, The School of Medicine and Public Health, the University of Newcastle, Newcastle, NSW, Australia.

[5] Medical and Health Division, Electricity Generating Authority of Thailand, Nonthaburi

[6] Hunter Medical Research Institute, Newcastle, NSW, Australia

[7] Division of Cardiology, Department of Medicine, Faculty of Medicine, Ramathibodi Hospital, Mahidol University, Bangkok, Thailand

Supplementary Tables

Table 1 Relative Variance Increase (RVI), Fraction of Missing Information (FMI) and Relative efficiency based on the final GSEM model

Table 2 Characteristics of participants enrolled in the first EGAT cohort with and without complete data

Table 3 Factors associated with CVEs Outcome: univariate and multivariate analyses

Table 4 Factors associated with diabetes, hypertension, and serum creatinine: Multiple mediation models

Table 5 Example of using the forward and backward carry over methods for participants who had inconsistent missing and the outlier data

Table 6 Imputed variables and their predictors in multiple imputations models

Table 7 Possible causal models based on serial multiple mediator models of pathway A (SMMMA)

Table 8 Possible causal models based on serial multiple mediators of pathway B (SMMMB)

Supplementary Figures

Figure 1 Causal pathway diagrams of NLR and CVEs with their coefficients

A) Pathway A: NLR → (DM → Cr → HT) → CVEs

B) Pathway B: NLR →(DM → HT→ Cr) → CVEs

Figure 2 Causal pathway diagrams of NLR and CVEs: Parallel multiple mediation models

A) Pathway A: NLR → (DM → Cr → HT) → CVEs

B) Pathway B: NLR →(DM → HT→ Cr) → CVEs

Appendices

Appendix A STATA COMMANDS USED FOR MULTIPLE IMPUTATIONS

Appendix B STATA COMMANDS FOR MULTIPLE MEDIATION ANALYSIS

Supplementary Table 1 Relative Variance Increase (RVI), Fraction of Missing Information (FMI) and Relative efficiency based on the final GSEM

Variables	RVI*	FMI*	Relative efficiency	Total variance
DM Model				
NLR	0.3233	0.2452	0.9976	0.0020
Education [a]				
≤ High school	0.0886	0.0815	0.9992	0.0107
Vocational/Diploma	0.0869	0.0800	0.9992	0.0100
History of taking NSAIDs	0.0765	0.0711	0.9993	0.0075
Dyslipidemia				
well-controlled	0.1722	0.1473	0.9985	0.0552
poorly-controlled	0.1267	0.1127	0.9989	0.0338
BMI [b]				
Underweight	0.5465	0.3549	0.9965	0.0832
Overweight/obese	0.1815	0.1540	0.9985	0.0078
WHR	0.3057	0.2349	0.9977	0.0102
Creatinine Model				
NLR	0.4180<br>(0.4382)	0.2960<br>(0.3059)	0.9970<br>(0.9969)	0.0002<br>(0.0001)
Well-controlled HT [c]	0.1145	0.1029	0.9989	0.0003
Poorly-controlled HT [c]	0.5821	0.3696	0.9963	0.0002
DM	0.2596<br>(0.2478)	0.2068<br>(0.1992)	0.9979<br>(0.9980)	0.0002<br>(0.0002)
Female	0.2297<br>(0.2210)	0.1873<br>(0.1816)	0.9981<br>(0.9982)	0.0002<br>(0.0002)
Uric acid	0.5082<br>(0.5022)	0.3385<br>(0.3358)	0.9966<br>(0.9967)	0.0000<br>(0.0000)
Anemia	0.5379<br>(0.5383)	0.3513<br>(0.3515)	0.9965<br>(0.9965)	0.0005<br>(0.0005)
Poorly-controlled HT Model				
NLR	0.2619<br>(0.2812)	0.2082<br>(0.2202)	0.9979<br>(0.9978)	0.0022<br>(0.0023)
DM	0.2362<br>(0.2413)	0.1917<br>(0.1929)	0.9981<br>(0.9981)	0.0076<br>(0.0076)
Creatinine [d]	0.3432	0.2565	0.9974	0.0133
Age ≥ 60 y	0.2829<br>(0.2949)	0.2213<br>(0.2286)	0.9978<br>(0.9977)	0.0061<br>(0.0061)
Education [a]				
≤ High school	0.1808<br>(0.1769)	0.1535<br>(0.1507)	0.9985<br>(0.9985)	0.0105<br>(0.0105)
Vocational/Diploma	0.1480<br>(0.1483)	0.1292<br>(0.1294)	0.9987<br>(0.9987)	0.0084<br>(0.0085)

Variables	RVI*	FMI*	Relative efficiency	Total variance
Poorly-controlled HT Model (cont'd)				
Income [e]				
Middle income	0.4632	0.3179	0.9968	0.0075
	(0.4750)	(0.3235)	(0.9968)	(0.0076)
High income	0.4731	0.3226	0.9968	0.0106
	(0.4718)	(0.3219)	(0.9968)	(0.0106)
Alcohol consumption	0.2114	0.1750	0.9983	0.0060
	(0.2349)	(0.1909)	(0.9981)	(0.0049)
History of taking NSAIDs	0.1037	0.0941	0.9991	0.0071
	(0.1037)	(0.0941)	(0.9991)	(0.0071)
Dyslipidemia				
well-controlled	0.2379	0.1928	0.9981	0.0689
	(0.2381)	(0.1929)	(0.9981)	(0.0679)
poorly-controlled	0.1725	0.1475	0.9985	0.0341
	(0.1761)	(0.1501)	(0.9985)	(0.0338)
BMI [b]				
Underweight	0.2527	0.2024	0.9979	0.0390
	(0.2475)	(0.1991)	(0.9980)	(0.0389)
Overweight/Obese	0.2778	0.2182	0.9978	0.0068
	(0.2724)	(0.2148)	(0.9979)	(0.0068)
Exercise [c]				
1-2 times/week [f]	0.3661	0.2691	0.9973	0.0118
≥ 3 times/week [f]	0.4037	0.2888	0.9971	0.0086
WHR	0.2371	0.1923	0.9981	0.0069
	(0.2404)	(0.1944)	(0.9981)	(0.0069)
Uric acid [c]	0.7102	0.4173	0.9958	0.0006
Well-controlled HT Model				
NLR	0.2031	0.1693	0.9983	0.0026
	(0.2006)	(0.1675)	(0.9983)	(0.0026)
DM	0.1131	0.1018	0.9989	0.0104
	(0.1144)	(0.1028)	(0.9989)	(0.0104)
Creatinine [d]	0.1744	0.1489	0.9985	0.0135
Age ≥ 60 y	0.0772	0.0717	0.9992	0.0087
	(0.0804)	(0.0745)	(0.9993)	(0.0088)
Education [a]				
≤ High School	0.0902	0.0828	0.9992	0.0105
	(0.0896)	(0.0824)	(0.9992)	(0.0152)
Vocational/Diploma	0.0698	0.0698	0.9993	0.0117
	(0.0699)	(0.0654)	(0.9993)	(0.0117)
Income [e]				
Middle income	0.2679	0.2120	0.9979	0.0098
	(0.2654)	(0.2104)	(0.9979)	(0.0098)
High income	0.2639	0.2095	0.9979	0.0137
	(0.2586)	(0.2062)	(0.9979)	(0.0137)

Variables	RVI*	FMI*	Relative efficiency	Total variance
Well-controlled HT Model (cont'd)				
Alcohol consumption	0.0961	0.0878	0.9991	0.0081
	(0.1003)	(0.0913)	(0.9991)	(0.0081)
History of taking	0.0358	0.0346	0.9997	0.0089
NSAIDs	(0.0368)	(0.0355)	(0.9996)	(0.0089)
Dyslipidemia				
well-controlled	0.0911	0.0836	0.9992	0.0815
	(0.0910)	(0.0835)	(0.9992)	(0.0808)
poorly-controlled	0.0671	0.0629	0.9994	0.0502
	(0.0656)	(0.0617)	(0.9994)	(0.0498)
BMI [b]				
Underweight	0.1217	0.1087	0.9989	0.0843
	(0.1244)	(0.1108)	(0.9989)	(0.0843)
Overweight/Obese	0.0964	0.0881	0.9991	0.0093
	(0.0965)	(0.0881)	(0.9991)	(0.0093)
Exercise [c]				
1-2 times/week [f]	0.2428	0.1959	0.9980	0.0158
≥ 3 times/week [f]	0.3223	0.2446	0.9976	0.0117
WHR	0.1513	0.1317	0.9987	0.0119
	(0.1483)	(0.1294)	(0.9987)	(0.0119)
Uric acid [c]	0.3105	0.2378	0.9976	0.0008
Outcome CVEs Model				
NLR	1.5482	0.6105	0.9939	0.0123
Well-controlled HT	0.2289	0.1868	0.9981	0.0786
Poorly-controlled HT	0.5109	0.3397	0.9966	0.0631
Creatinine	0.4543	0.3137	0.9969	0.0173
DM	0.2251	0.1843	0.9982	0.0378
History of taking NSAIDs	0.0156	0.0154	0.9998	0.0334
Age ≥ 60 y	0.0108	0.0106	0.9999	0.1822
Smoking	0.2206	0.1813	0.9982	0.0569

The number in parentheses illustrated the values of pathway B. All values in DM model were the same for both pathways.

a, compared with Bachelor or higher degree; b, compared with normal weight; c, only used in pathway B; d, used in pathway A only;

e, compared with low income (< 20,000 Baht/month); middle income (20,000-49,999 Baht/month); high income (≥ 50,000 Baht/month);

f, compared with ≤ 1 time/week

DM, diabetes mellitus; NLR, neutrophil lymphocyte ratio; BMI, body mass index; WHR, waist hip ratio.

* The largest FMI coefficient and the average RVI which quantifies uncertainty of the values estimated from multiple imputations

[RIV=$(1 + M^{-1})B/U$ and FMI = $\frac{RIV+2/(V+3)}{RIV+1}$; M: number of imputations, B: between variance imputation, U: within variance imputation, V: degrees of freedom]

Supplementary Table 2 Characteristics of participants enrolled in the first EGAT cohort with and without completed data

Characteristics	Without completed data	With completed data by imputation
	Percentage	percentage
Education		
≤ High School	26.1	27.0
Vocational/Diploma	30.4	30.7
≥ Bachelor	43.5	42.2
Income (Baht/month)		
< 20,000	26.0	32.6
20,000 – 49,999	35.9	33.1
≥ 50,000	38.1	34.3
Marital status		
Single	5.8	5.3
Married	82.6	81.4
Widowed/ separate/divorce	11.6	13.3
Smoking		
Non-smoker	45.2	42.7
Ex-smoker	38.3	35.6
Current smoker	16.5	21.7
Alcohol Drinking		
Non-drinker	45.6	45.3
Ex-drinker	24.9	28.2
Current drinker	29.6	26.5
Exercise		
None	17.91	18.3
1 – 2 times/week	15.96	15.3
≥ 3 times/week	66.13	66.4
Lipid lowering medication		
Yes	47.6	47.3
No	52.4	52.7

Characteristics	Without completed data		With completed data by imputation	
	mean	SD	mean	SD
Height, cm	163.0	7.2	163.0	7.2
Body weight, kg	64.9	10.2	64.9	10.4
Waist circumference, cm	89.8	9.0	90.0	9.2
Hip circumference, cm	96.4	6.5	96.3	6.7
SBP, mmHg	133.1	19.8	133.9	20.0
DBP, mmHg	81.0	11.6	80.6	11.7
Neutrophil (%)	56.9	8.9	56.8	9.0
Lymphocyte (%)	34.7	7.8	34.8	7.9
Platelets, x10^3 cells/mL	251.3	63.9	200.5	101.9
Hemoglobin, g/dL	13.8	1.6	13.7	1.6
FPG, mg/dL	101.4	29.8	103.2	29.8
TC, mg/dL	226.6	45.2	223.5	45.2
TG, mg/dL	145.7	93.8	149.2	91.4
LDL, mg/dL	147.4	41.0	144.7	41.0
HDL, mg/dL	55.6	14.6	56.0	14.8
Creatinine, mg/dL	1.0	0.4	1.1	0.5
Uric acid, mg/dL	6.1	1.5	6.2	1.6

SBP, systolic blood pressure; DBP, diastolic blood pressure; FPG, fasting plasma glucose; TC, total cholesterol; TG, triglyceride; LDL, low-density lipoprotein; HDL, high-density lipoprotein

Supplementary Table 3 Factors associated with CVEs: Univariate and Multivariate Analysis

Factors	B	SE	T	P	95%CI
Univariate GSEM					
NLR	0.29	0.09	3.30	0.001	0.12, 0.48
Diabetes mellitus	0.99	0.18	5.46	<0.001	0.63, 1.34
Creatinine	0.41	0.12	3.45	0.001	0.18, 0.64
Poorly-controlled HT	1.02	0.24	4.18	<0.001	0.54, 1.49
Well-controlled HT	0.94	0.26	3.61	<0.001	0.43, 1.46
Age	0.15	0.01	14.64	<0.001	0.13, 0.18
Sex (Female)	-0.62	0.33	-1.88	0.06	-1.26, 0.03
Education					
≤ High school vs. ≥ Bachelor	0.42	0.31	1.37	0.17	-0.18, 1.03
Vocational/Diploma vs. ≥ Bachelor	0.31	0.29	1.03	0.30	-0.28, 0.89
Income					
Low vs. High income	0.97	0.34	2.82	0.005	0.29, 1.64
Middle vs. High income	0.34	0.29	1.17	0.242	-0.23, 0.92
Marital status					
Married vs. Single	9.06	6.77	1.34	0.18	-4.37, 22.49
Widowed/Separate/Divorce vs. Single	9.28	6.79	1.37	0.18	-4.20, 22.77
BMI					
Underweight vs. Normal	-1.63	3.12	-0.52	0.602	-7.82, 4.55
Overweight and obese vs. Normal	-0.14	0.21	-0.66	0.509	-0.55, 0.27
WHR (high)	6.00	2.15	2.79	0.006	1.77, 10.24
History of taking NSAIDs	0.65	0.19	3.31	0.001	0.27, 1.03
Smoker	1.06	0.23	4.54	<0.001	0.59, 1.51
Alcohol drinker	0.96	0.25	3.91	<0.001	0.48, 1.45
Exercise					
1 – 2 times/week	-0.69	0.36	-1.95	0.05	-1.39, 0.01
≥ 3 times/week	-0.45	0.34	-1.32	0.19	-1.11, 0.22
Dyslipidemia	0.81	0.36	2.22	0.03	0.09, 1.53
Uric acid	0.17	0.08	2.09	0.04	0.01, 0.34
Hemoglobin	-0.06	0.07	-0.81	0.417	-0.19, 0.08
Anemia	0.78	0.31	2.53	0.012	0.17, 1.39
Multivariate GSEM					
NLR	0.2246	0.1109	2.03	0.044	0.0064, 0.4429
Diabetes	0.4649	0.1945	2.39	0.017	0.0836, 0.8462
Creatinine	0.4697	0.1315	3.57	<0.001	0.2115, 0.7278
Poorly-controlled HT	0.5615	0.2512	2.24	0.026	0.0684, 1.0545
Well-controlled HT	0.1289	0.2804	0.46	0.646	-0.4210, 0.6787
History of taking NSAIDs	0.6986	0.1829	3.82	<0.001	0.3401, 1.0570
Age ≥ 60 y	2.3014	0.4268	5.39	<0.001	1.4649, 3.1380
Smoker	0.7920	0.2385	3.32	0.001	0.3243, 1.2598

Low income, <20,000 Baht/month; Middle income, 20,000-49,999 Baht/month; High income, ≥ 50,000 Baht/month; underweight, normal weight, overweight and obesity when the participants had BMI < 18.5, 18.5-22.9, 23-24.9 and at least 25 Kg/m2, respectively; high WHR ≥ 0.9 in male or ≥ 0.85 in female; Smoker, current plus ex-smoker; Alcohol drinker (current plus quit drinker).

b, coefficient; SE, standard error; t, t-test; P, P-value; CI, confidence interval; GSEM, generalized structural equation model; NLR, neutrophil lymphocyte ratio; BMI, body mass index; WHR, waist hip ratio.

Supplementary Table 4 Factors associated with diabetes, hypertension, and serum creatinine: Multiple mediation models

Equation	Factors	B	SE	t	P-value	95% CI
NLR → Diabetes Mellitus	NLR	0.1213	0.0448	2.71	0.007	0.0334, 0.2092
	Education [a]					
	High school	0.4138	0.1033	4.00	<0.001	0.2113, 0.6164
	Vocational/Diploma	0.2779	0.1001	2.78	0.006	0.0817, 0.4741
	History of taking NSAIDs	0.4948	0.0868	5.70	<0.001	0.3247, 0.6649
	Dyslipidemia					
	well-controlled	0.7674	0.2350	3.26	0.001	0.3066, 1.2282
	poorly-controlled	0.1792	0.1839	0.97	0.330	-0.1814, 0.5398
	Underweight [b]	-0.8338	0. 2884	-2.89	0.004	-1.4000, -0.2676
	Obese [b]	0.1603	0.0885	1.81	0.070	-0.0133, 0.3338
	WHR (high) [c]	0.5887	0.1008	5.84	<0.001	0.3910, 0.7863
NLR → Creatinine	NLR	0.0294	0.0122	2.40	0.017	0.0054, 0.0534
	Diabetes mellitus	0.0539	0.0147	3.67	<0.001	0.0252, 0.0828
	Female	-0.1449	0.0134	-10.84	<0.001	-0.1711, -0.1187
	Uric acid	0.0659	0.0058	11.37	<0.001	0.0546, 0.0773
	Anemia	0.0906	0.0226	4.00	<0.001	0.0462, 0.1350
NLR → Poorly-controlled HT	NLR	0.1213	0.0474	2.56	0.011	0.0283, 0.2143
	Diabetes mellitus	0.5122	0.0871	5.88	<0.001	0.3414, 0.6830
	Creatinine	0.3282	0.1145	2.87	0.004	0.1036, 0.5528
	Age > 60 y	0.5979	0.0781	7.66	<0.001	0.4447, 0.7510
	Education [a]					
	High school	0.3863	0.1024	3.77	<0.001	0.1855, 0.5871
	Vocational/Diploma	0.3234	0.0921	3.51	<0.001	0.1429, 0.5039
	Income [d]					
	Middle income	-0.1041	0.0867	-1.20	0.230	-0.2743, 0.0663
	High income	-0.1466	0.1030	-1.42	0.155	-0.3488, 0.0555
	Alcoholic drinker [e]	0.2848	0.0777	3.66	<0.001	0.1323, 0.4372
	Exercise [f]					
	1-2 / week	-0.1787	0.1088	-1.64	0.101	-0.3921, 0.0346
	≥ 3 / week	-0.0207	0.0922	-0.22	0.823	-0.2015, 0.1602
	History of taking NSAIDs	0.5345	0.0842	6.35	<0.001	0.3695, 0.6995
	Dyslipidemia					
	well-controlled	0.3618	0.2629	1.38	0.169	-0.1537, 0.8773
	poorly-controlled	0.4038	0.1849	2.18	0.029	0.0413, 0.7663
	Underweight [b]	-0.4584	0.1973	-2.32	0.020	-0.8453, -0.0715
	Obese [b]	0.57283	0.0826	6.93	<0.001	0.4108, 0.7349
	WHR (high) [c]	0.2999	0.0832	3.60	<0.001	0.1367, 0.4630
NLR → Well-controlled HT	NLR	0.2448	0.0513	4.77	<0.001	0.1442, 0.3454
	Diabetes mellitus	0.4609	0.1020	4.52	<0.001	0.2609, 0.6609
	Creatinine	0.2786	0.1154	2.41	0.016	0.0524, 0.5049
	Age > 60 y	0.7558	0.0939	8.05	<0.001	0.5717, 0.9399

Education [a]					
High school	-0.0944	0.1233	-0.77	0.444	-0.3359, 0.1472
Vocational/Diploma	0.0989	0.1081	0.91	0.360	-0.1130, 0.3108
Income [d]					
Middle income	-0.1971	0.0993	-1.98	0.047	-0.3919, -0.0023
High income	-0.4032	0.1177	-3.42	0.001	-0.6340, -0.1723
Alcoholic drinker [e]	-0.0049	0.0903	-0.06	0.956	-0.1819, 0.1720
Exercise [f]					
1-2 / week	-0.2489	0.1255	-1.98	0.048	-0.4951, -0.0027
≥ 3 / week	-0.0755	0.1086	-0.70	0.487	-0.2884, 0.1375
History of taking NSAIDs	0.9675	0.0947	10.22	<0.001	0.7818, 1.1529
Dyslipidemia					
well-controlled	1.2229	0.2857	4.28	<0.001	0.6629, 1.7828
poorly-controlled	0.3619	0.2243	1.61	0.107	-0.0776, 0.8015
Underweight [b]	-0.9161	0.2905	-3.15	0.002	-1.4855, -0.3466
Obese [b]	0.6237	0.0965	6.46	<0.001	0.4345, 0.8128
WHR (high) [c]	0.4921	0.1092	4.50	<0.001	0.2779, 0.7062

a, compared with Bachelor or higher degree; b, compared with normal weight; underweight, normal weight, overweight and obesity when the participants had BMI < 18.5, 18.5-22.9, 23-24.9 and at least 25 Kg/m2, respectively; c, high WHR ≥ 0.9 in male or ≥ 0.85 in female; d, compared with low income (< 20,000 Baht/month); middle income, 20,000-49,999 Baht/month; high income, ≥ 50,000 Baht/month; e, Alcohol drinker (current plus quit drinker); f, compared with ≤ 1 time/week.

b, coefficient; SE, standard error; t, t-test; P, P-value; CI, confidence interval; NLR, neutrophil lymphocyte ratio; BMI, body mass index; WHR, waist hip ratio.

Supplementary Table 5 Example of using the forward and backward carry over methods for participants who had inconsistent missing and the outlier data

Gender variable

No.Participant	1	2		1	2
EGAT1/1	Female	Male		Female	Male
EGAT1/2	Female	Male		Female	Male
EGAT1/3	Female	**Missing**	⇒	Female	**Male**
EGAT1/4	**Male**	Male		**Female**	Male
EGAT1/5	Female	Male		Female	Male

Height variable

No.Participant	1	2		1	2
EGAT1/1	160	180		160	180
EGAT1/2	160	**Missing**		160	**180**
EGAT1/3	**66**	180	⇒	**160**	180
EGAT1/4	160	**80**		160	**180**
EGAT1/5	160	180		160	180

Education variable

No.Participant	1	2		1	2
EGAT1/1	≤ High school	Diploma		≤High school	Diploma
EGAT1/2	Bachelor	Diploma		Bachelor	Diploma
EGAT1/3	Bachelor	**Missing**	⇒	Bachelor	**Diploma**
EGAT1/4	**≤ High school**	Diploma		**Bachelor**	Diploma
EGAT1/5	Bachelor	Diploma		Bachelor	Diploma

Marital status variable

No.Participant	1	2		1	2
EGAT1/1	Single	Single		Single	Single
EGAT1/2	Married	Single		Married	Single
EGAT1/3	Married	**Missing**	⇒	Married	**Single**
EGAT1/4	Divorce	Single		Divorce	Single
EGAT1/5	**Single**	Single		**Divorce**	Single

Supplementary Table 6 Imputed variables and their predictors in multiple imputation models

Imputed variable	Predictors																											Outcome		No. predictors	Equation
	Sex	Age	Education	Income	Mstatus	Smoking	Alcohol	Exercise	Weight	Height	Waist	Hip	SBP	DBP	NSAIDs	Lipid Drug	Glucose	Cholesterol	HDL	LDL	Triglyceride	Uric Acid	Creatinine	Neutrophils	Lymphocytes	Platelets	Hemoglobin	E_Composit	D_Composit		
Education	√	√	√	√	√	√	√	√	√	√	√	√	√	√	√	√	√	√	√		√	√	√					√	√	24	intreg
Income	√	√	√	√	√	√	√	√	√	√	√	√	√	√	√	√	√	√	√		√		√					√	√	23	ologit
Marital status	√	√	√	√	√	√	√	√	√	√	√	√																√	√	14	intreg
Smoking	√	√	√	√	√	√	√	√	√	√	√	√	√	√	√		√	√	√		√	√	√	√	√	√	√	√	√	27	intreg
Alcohol	√	√	√	√	√	√	√	√	√	√	√	√	√	√	√		√	√	√		√	√		√	√	√	√	√	√	26	intreg
Exercise	√	√	√	√	√	√	√	√	√	√	√	√	√	√	√		√	√	√		√	√	√	√	√	√	√	√	√	27	ologit
Height	√	√							√	√	√	√	√	√																8	regress
Weight	√	√	√	√		√	√	√	√	√	√	√	√	√		√	√	√	√		√	√	√	√	√	√		√	√	25	regress
Waist	√	√	√	√		√	√	√	√	√	√	√	√	√		√	√	√	√		√	√	√	√	√	√		√	√	25	regress
Hip	√	√	√	√		√	√	√	√	√	√	√	√	√		√	√	√	√		√	√	√	√	√	√		√	√	25	regress
SBP	√	√	√	√		√	√	√	√	√	√	√	√	√	√	√	√	√	√		√	√	√	√	√	√	√	√	√	27	regress
DBP	√	√	√	√		√	√	√	√	√	√	√	√	√	√	√	√	√	√		√	√	√	√	√	√	√	√	√	27	regress
Lipid Drug	√	√	√	√				√	√	√	√	√	√	√		√	√	√	√		√			√	√	√		√	√	21	logit
Glucose	√	√	√	√		√	√	√	√	√	√	√	√	√		√	√	√	√		√	√	√	√	√	√	√	√	√	26	intreg
Cholesterol	√	√	√	√		√	√	√	√	√	√	√	√	√		√	√	√	√		√	√	√	√	√	√		√	√	25	intreg
HDL	√	√	√	√		√	√	√	√	√	√	√	√	√		√	√	√	√		√	√	√	√	√	√		√	√	25	intreg
LDL	√	√	√	√		√	√	√	√	√	√	√	√	√		√	√			√	√	√	√	√	√	√		√	√	24	intreg
Triglyceride	√	√	√	√		√	√	√	√	√	√	√	√	√		√	√	√	√		√	√	√	√	√	√		√	√	25	intreg
Uric Acid	√	√	√	√		√	√	√	√	√	√	√	√				√	√	√		√	√	√					√	√	20	intreg
Creatinine	√	√	√	√		√	√	√	√	√	√	√	√	√	√		√			√	√	√	√	√	√	√	√	√	√	24	intreg
Neutrophils	√	√				√	√	√	√	√	√	√	√	√	√	√	√	√	√	√	√	√	√	√	√	√	√	√	√	26	intreg
Lymphocytes	√	√				√	√	√	√	√	√	√	√	√	√	√	√	√	√	√	√	√	√	√	√	√	√	√	√	26	intreg
Platelets	√	√				√	√	√	√	√	√	√	√	√	√	√	√	√	√	√	√	√	√	√	√	√	√	√	√	26	intreg
Hemoglobin	√	√				√	√	√	√	√	√	√	√	√	√	√	√	√	√	√	√	√	√	√	√	√	√	√	√	26	intreg

Supplementary Table 7 Possible causal models based on serial multiple mediator models of pathway A (SMMMA)

SMMMA1									
1) a_1b_1	NLR	→	DM				→		CVEs
2) $a_1b_2b_8$	NLR	→	DM	→	well-controlled HT		→		CVEs
3) $a_1b_3b_9$	NLR	→	DM	→	poorly-controlled HT		→		CVEs
4) $a_1b_4b_5$	NLR	→	DM	→	Creatinine		→		CVEs
5) $a_1b_4b_6b_8$	NLR	→	DM	→	Creatinine	→	well-controlled HT	→	CVEs
6) $a_1b_4b_7b_9$	NLR	→	DM	→	Creatinine	→	poorly-controlled HT	→	CVEs
SMMMA2									
7) a_2b_5	NLR	→	Creatinine				→		CVEs
8) $a_2b_6b_8$	NLR	→	Creatinine	→	well-controlled HT		→		CVEs
9) $a_2b_7b_9$	NLR	→	Creatinine	→	poorly-controlled HT		→		CVEs
SMMMA3									
10) a_3b_8	NLR	→	well-controlled HT			→			CVEs
SMMA4									
11) a_4b_9	NLR	→	poorly-controlled HT			→			CVEs

NLR, neutrophil-lymphocyte ratio; CVEs, cardiovascular events; DM, Diabetes Mellitus; HT, hypertension

SMMA1: Serial multiple mediator model of pathway A with DM as initial mediator

SMMA2: Serial multiple mediator model of pathway A with creatinine as initial mediator

SMMA3 and SMMMA4: Serial multiple mediator model of pathway A with well- and poorly-controlled HT as initial mediators, respectively.

Supplementary Table 8 Possible causal models based on serial multiple mediator models of pathway B (SMMMB)

SMMMB1									
1) a_1b_1	NLR	→	DM					→	CVEs
2) $a_1b_2b_9$	NLR	→	DM	→	Creatinine			→	CVEs
3) $a_1b_3b_7$	NLR	→	DM	→	well-controlled HT			→	CVEs
4) $a_1b_4b_8$	NLR	→	DM	→	poorly-controlled HT			→	CVEs
5) $a_1b_3b_5b_9$	NLR	→	DM	→	well-controlled HT	→	Creatinine	→	CVEs
6) $a_1b_4b_6b_9$	NLR	→	DM	→	poorly-controlled HT	→	Creatinine	→	CVEs
SMMMB2									
7) a_4b_9	NLR	→	Creatinine					→	CVEs
SMMMB3									
8) a_2b_7	NLR	→	well-controlled HT					→	CVEs
9) $a_2b_5b_9$	NLR	→	well-controlled HT	→	Creatinine			→	CVEs
SMMMB4									
10) a_3b_8	NLR	→	poorly-controlled HT					→	CVEs
11) $a_3b_6b_9$	NLR	→	poorly-controlled HT	→	Creatinine			→	CVEs

NLR, neutrophil-lymphocyte ratio; CVEs, cardiovascular events; DM, Diabetes Mellitus; HT, hypertension;

SMMB1: Serial multiple mediator model of pathway B with DM as initial mediator;

SMMB2: Serial multiple mediator model of pathway B with creatinine as initial mediator;

SMMB3 and SMMMB4: Serial multiple mediator model of pathway B with well- and poorly-controlled HT as initial mediators, respectively.

A) Pathway A: NLR→ (DM → Cr→ HT)→ CVEs

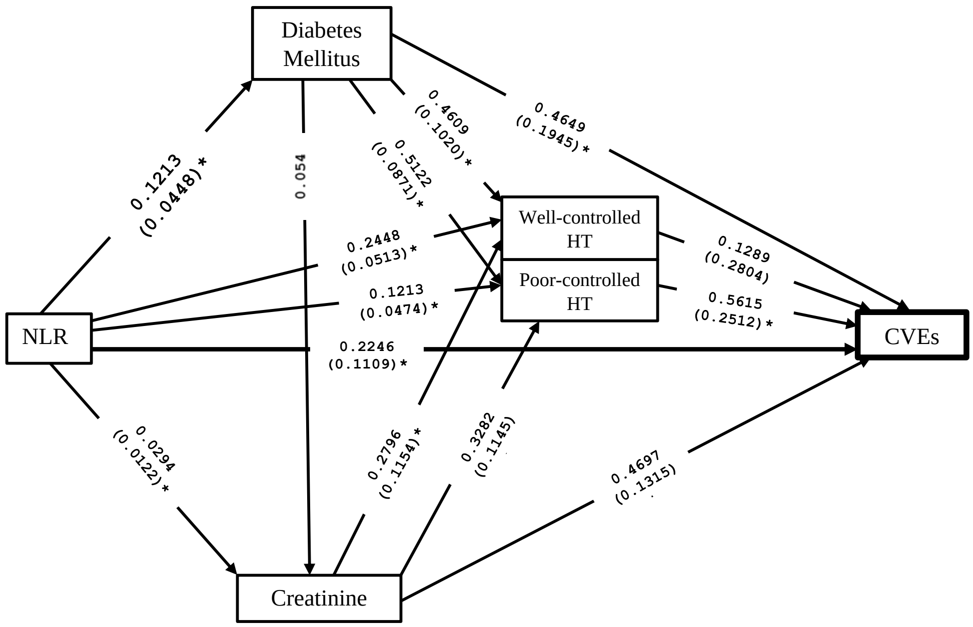

B) Pathway B: NLR →(DM → HT→ Cr)→CVEs

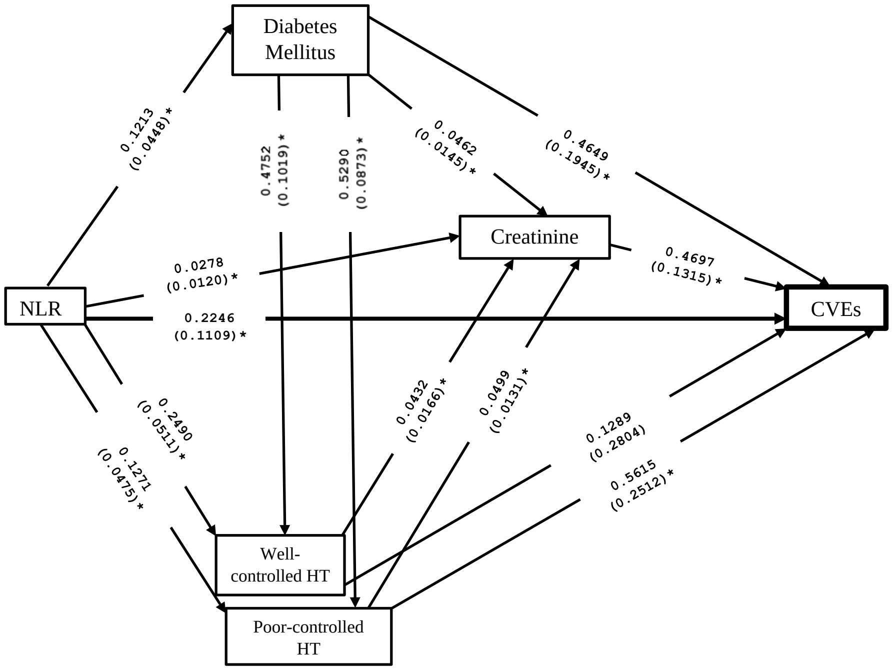

Supplementary Figure 1 Causal pathway diagrams of NLR and CVEs with their coefficients

Values in parentheses = standard error; values outside parentheses = path coefficient; *, P<0.05.

NLR, neutrophil-lymphocyte ratio; CVEs, cardiovascular events

A) Pathway A: NLR→ (DM → Cr→ HT)→ CVEs

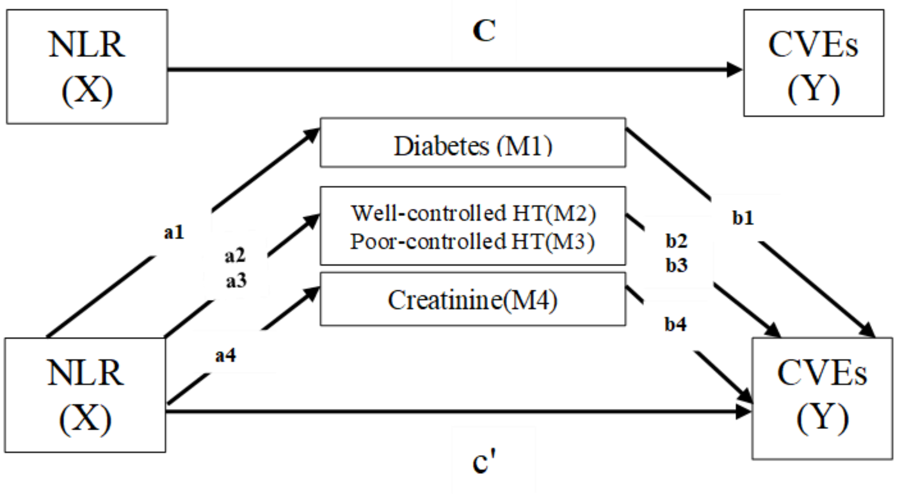

B) Pathway B: NLR →(DM → HT→ Cr)→CVEs

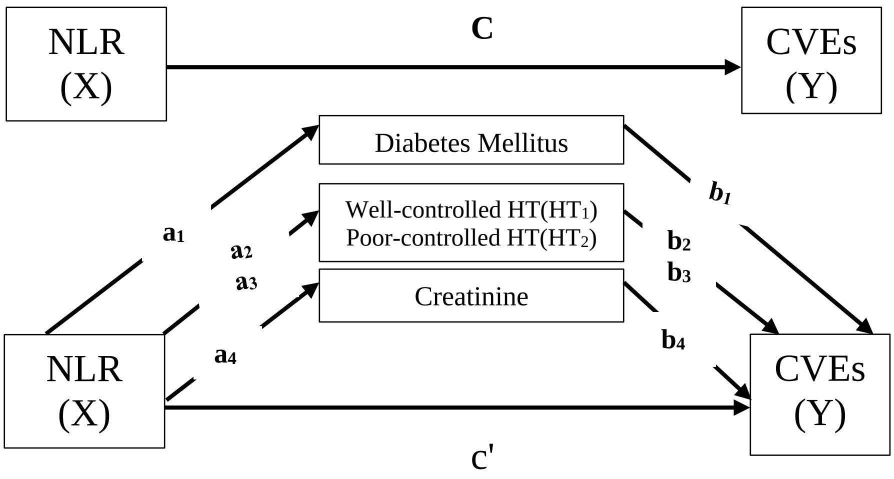

Supplementary Figure 2 Causal pathway diagrams of NLR and CVEs: Parallel multiple mediation models

NLR, neutrophil-lymphocyte ratio; DM, diabetes; Cr, creatinine; CVEs, cardiovascular events

APPENDIX A

STATA COMMANDS USED FOR MULTIPLE IMPUTATIONS

a) Multiple imputations

```
set matsize 11000
mi set mlong
mi register imputed neu* lymph* plt* hb* edu* inc* mstatus* smk* alc* exe* height weight* waist* hip* sbp*
        dbp* glu* chol* hdl* tri* ldl* uric* cre* drfat_sum*
mi register regular sex age* fm_fat fm_dm fm_ht event_composit1 d_composit1 drnsaid*
mi impute chained ///
   (intreg,ll(zneu13_l) ul(zneu13_u)        include (i.sex  age* height weight* waist* hip* i.drnsaid*
         xneu* xlymph* xplt* xhb* event_composit1 d_composit1 i.drfat_sum* xsmk* xalc* i.exe* sbp* dbp*
         xglu* xchol* xhdl* xtri* xuric* xcre*)) xneu13 ///
   (intreg,ll(zneu14_l) ul(zneu14_u)        include (i.sex  age* height weight* waist* hip* i.drnsaid*
         xneu* xlymph* xplt* xhb* event_composit1 d_composit1 i.drfat_sum* xsmk* xalc* i.exe* sbp* dbp*
         xglu* xchol* xhdl* xtri* xuric* xcre*)) xneu14 ///
   (intreg,ll(zneu15_l) ul(zneu15_u)        include (i.sex  age* height weight* waist* hip* i.drnsaid*
         xneu* xlymph* xplt* xhb* event_composit1 d_composit1 i.drfat_sum* xsmk* xalc* i.exe* sbp* dbp*
         xglu* xchol* xhdl* xtri* xuric* xcre*)) xneu15 ///
   (intreg,ll(zlymph13_l) ul(zlymph13_u)    include (i.sex  age* height weight* waist* hip* i.drnsaid*
         xneu* xlymph* xplt* xhb* event_composit1 d_composit1 i.drfat_sum* xsmk* xalc* i.exe* sbp* dbp*
         xglu* xchol* xhdl* xtri* xuric* xcre*)) xlymph13 ///
   (intreg,ll(zlymph14_l) ul(zlymph14_u)    include (i.sex  age* height weight* waist* hip* i.drnsaid*
         xneu* xlymph* xplt* xhb* event_composit1 d_composit1 i.drfat_sum* xsmk* xalc* i.exe* sbp* dbp*
         xglu* xchol* xhdl* xtri* xuric* xcre*)) xlymph14 ///
   (intreg,ll(zlymph15_l) ul(zlymph15_u)    include (i.sex  age* height weight* waist* hip* i.drnsaid*
         xneu* xlymph* xplt* xhb* event_composit1 d_composit1 i.drfat_sum* xsmk* xalc* i.exe* sbp* dbp*
         xglu* xchol* xhdl* xtri* xuric* xcre*)) xlymph15 ///
   (intreg,ll(zplt13_l) ul(zplt13_u)        include (i.sex  age* height weight* waist* hip* i.drnsaid*
         xneu* xlymph* xplt* xhb* event_composit1 d_composit1 i.drfat_sum* xsmk* xalc* i.exe* sbp* dbp*
         xglu* xchol* xhdl* xtri* xuric* xcre*)) xplt13 ///
```

```
(intreg,ll(zplt14_l) ul(zplt14_u)         include (i.sex  age* height weight* waist* hip* i.drnsaid*
      xneu* xlymph* xplt* xhb* event_composit1 d_composit1 i.drfat_sum* xsmk* xalc* i.exe* sbp* dbp*
      xglu* xchol* xhdl* xtri* xuric* xcre*)) xplt14 ///
(intreg,ll(zplt15_l) ul(zplt15_u)         include (i.sex  age* height weight* waist* hip* i.drnsaid*
      xneu* xlymph* xplt* xhb* event_composit1 d_composit1 i.drfat_sum* xsmk* xalc* i.exe* sbp* dbp*
      xglu* xchol* xhdl* xtri* xuric* xcre*)) xplt15 ///
(intreg,ll(zhb13_l) ul(zhb13_u)           include (i.sex  age* height weight*             i.drnsaid*
      xneu* xlymph* xplt* xhb* event_composit1 d_composit1              xsmk* xalc* i.exe* sbp* dbp*
      xglu* xchol* xhdl* xtri* xuric* xcre*)) xhb13 ///
(intreg,ll(zhb14_l) ul(zhb14_u) include (i.sex  age* height weight* i.drnsaid* xneu* xlymph* xplt* xhb*
      event_composit1 d_composit1 xsmk* xalc* i.exe* sbp* dbp* xglu* xchol* xhdl* xtri* xuric* xcre*))
      xhb14 ///
(intreg,ll(zhb15_l) ul(zhb15_u) include (i.sex  age* height weight*             i.drnsaid* xneu* xlymph*
      xplt* xhb* event_composit1 d_composit1 xsmk* xalc* i.exe* sbp* dbp* xglu* xchol* xhdl* xtri*
      xuric* xcre*)) xhb15 ///
(intreg,ll(zedu12_l) ul(zedu12_u)  include (i.sex  age* height weight* waist* hip* i.drnsaid*
      event_composit1 d_composit1 xedu* i.inc* i.drfat_sum* xmstatus* xsmk* xalc* i.exe* sbp* dbp* xglu*
      xchol* xhdl* xtri* xuric* xcre* )) xedu12 ///
(intreg,ll(zedu13_l) ul(zedu13_u) include (i.sex  age* height weight* waist* hip* i.drnsaid*
      event_composit1 d_composit1 xedu* i.inc* i.drfat_sum* xmstatus* xsmk* xalc* i.exe* sbp* dbp* xglu*
      xchol* xhdl* xtri* xuric* xcre*)) xedu13 ///
(intreg,ll(zedu14_l) ul(zedu14_u)  include (i.sex  age* height weight* waist* hip* i.drnsaid*
      event_composit1 d_composit1 xedu* i.inc* i.drfat_sum* xmstatus* xsmk* xalc* i.exe* sbp* dbp* xglu*
      xchol* xhdl* xtri* xuric* xcre*)) xedu14 ///
(ologit,iterate(100) augmen      include (i.sex  age* height weight* waist* hip* i.drnsaid*
      event_composit1 d_composit1 xedu* i.inc* i.drfat_sum* xmstatus* xsmk* xalc* i.exe* sbp* dbp* xglu*
      xchol* xhdl* xtri* xcre*)) inc12 ///
(ologit,iterate(100) augmen       include (i.sex  age* height weight* waist* hip* i.drnsaid*
      event_composit1 d_composit1 xedu* i.inc* i.drfat_sum* xmstatus* xsmk* xalc* i.exe* sbp* dbp* xglu*
      xchol* xhdl* xtri*        xcre*)) inc13 ///
(ologit,iterate(100) augment include (i.sex  age* height weight* waist* hip* i.drnsaid* event_composit1
      d_composit1 xedu* i.inc* i.drfat_sum* xmstatus* xsmk* xalc* i.exe* sbp* dbp* xglu* xchol* xhdl*
      xtri* xcre*)) inc14 ///
(ologit,iterate(100) augmen      include (i.sex  age* height weight* waist* hip* i.drnsaid*
      event_composit1 d_composit1 xedu* i.inc* i.drfat_sum* xmstatus* xsmk* xalc* i.exe* sbp* dbp* xglu*
      xchol* xhdl* xtri*  xcre* )) inc15 ///
```

```
(intreg,ll(zmstatus12_l)   ul(zmstatus12_u) include (i.sex  age* weight* waist* hip* i.drnsaid*
      event_composit1 d_composit1 xedu* i.inc* xmstatus* xsmk* xalc* i.exe*)) xmstatus12 ///
(intreg,ll(zmstatus13_l)   ul(zmstatus13_u) include (i.sex  age* weight* waist* hip* i.drnsaid*
      event_composit1 d_composit1 xedu* i.inc* xmstatus* xsmk* xalc* i.exe*)) xmstatus13 ///
(intreg,ll(zmstatus14_l)   ul(zmstatus14_u) include (i.sex  age* weight* waist* hip* i.drnsaid*
      event_composit1 d_composit1 xedu* i.inc* xmstatus* xsmk* xalc* i.exe*)) xmstatus14 ///
(intreg,ll(zmstatus15_l)   ul(zmstatus15_u) include (i.sex  age* weight* waist* hip* i.drnsaid*
      event_composit1 d_composit1 xedu* i.inc* xmstatus* xsmk* xalc* i.exe*)) xmstatus15 ///
(intreg,ll(zsmk12_l)     ul(zsmk12_u)     include (i.sex  age* waist* weight* hip* i.drnsaid* xneu*
      xlymph* xplt* xhb* event_composit1 d_composit1 xedu* i.inc* xmstatus* xsmk* xalc* i.exe* sbp* dbp*
      xglu* xchol* xhdl* xtri* xuric* xcre*)) xsmk12 ///
(intreg,ll(zsmk13_l)     ul(zsmk13_u) include (i.sex  age* waist* weight* hip* i.drnsaid* xneu* xlymph*
      xplt* xhb* event_composit1 d_composit1 xedu* i.inc* xmstatus* xsmk* xalc* i.exe* sbp* dbp* xglu*
      xchol* xhdl* xtri* xuric* xcre* )) xsmk13 ///
(intreg,ll(zsmk14_l)     ul(zsmk14_u)     include (i.sex  age* waist* weight* hip* i.drnsaid* xneu*
      xlymph* xplt* xhb* event_composit1 d_composit1 xedu* i.inc* xmstatus* xsmk* xalc* i.exe* sbp* dbp*
      xglu* xchol* xhdl* xtri* xuric* xcre*)) xsmk14 ///
(intreg,ll(zsmk15_l)     ul(zsmk15_u)     include (i.sex  age* waist* weight* hip* i.drnsaid* xneu*
      xlymph* xplt* xhb* event_composit1 d_composit1 xedu* i.inc* xmstatus* xsmk* xalc* i.exe* sbp* dbp*
      xglu* xchol* xhdl* xtri* xuric* xcre* )) xsmk15 ///
(intreg,ll(zalc12_l)     ul(zalc12_u)     include (i.sex  age* waist* weight* hip* i.drnsaid* xneu*
      xlymph* xplt* xhb* event_composit1 d_composit1 xedu* i.inc* xmstatus* xsmk* xalc* i.exe* sbp* dbp*
      xglu* xchol* xhdl* xtri* xuric*)) xalc12 ///
(intreg,ll(zalc13_l)     ul(zalc13_u)  include (i.sex  age* waist* weight* hip* i.drnsaid* xneu* xlymph*
      xplt* xhb* event_composit1 d_composit1 xedu* i.inc* xmstatus* xsmk* xalc* i.exe* sbp* dbp* xglu*
      xchol* xhdl* xtri* xuric*)) xalc13 ///
(intreg,ll(zalc14_l)     ul(zalc14_u) include (i.sex  age* waist* weight* hip* i.drnsaid* xneu* xlymph*
      xplt* xhb* event_composit1 d_composit1 xedu* i.inc* xmstatus* xsmk* xalc* i.exe* sbp* dbp* xglu*
      xchol* xhdl* xtri* xuric*)) xalc14 ///
(intreg,ll(zalc15_l)     ul(zalc15_u)  include (i.sex  age* waist* weight* hip* i.drnsaid* xneu* xlymph*
      xplt* xhb* event_composit1 d_composit1 xedu* i.inc* xmstatus* xsmk* xalc* i.exe* sbp* dbp* xglu*
      xchol* xhdl* xtri* xuric*)) xalc15 ///
(ologit, iterate(100) augmen   include (i.sex  age* waist* weight* hip* i.drnsaid* xneu* xlymph* xplt*
      xhb* event_composit1 d_composit1 xedu* i.inc* xmstatus* xsmk* xalc* i.exe* sbp* dbp* xglu* xchol*
      xhdl* xtri* xuric* xcre*)) exe* ///
(regress,                      include (i.sex  age* height weight* waist* hip* sbp* dbp*)) height ///
```

```
(regress,                        include (i.sex  age* xedu*  xneu* xlymph* xplt* event_composit1
      d_composit1 i.inc* xsmk* xalc* i.exe* i.drfat_sum* height weight* waist* hip* sbp* dbp* xglu*
      xchol* xhdl* xtri* xuric* xcre* )) weight* ///
(regress,                      include (i.sex  age* xedu*  xneu* xlymph* xplt* event_composit1 d_composit1
      i.inc* xsmk* xalc* i.exe* i.drfat_sum* height weight* waist* hip* sbp* dbp* xglu* xchol* xhdl*
      xtri* xuric* xcre*)) waist* ///
(regress,                         include (i.sex  age* xedu*  xneu* xlymph* xplt* event_composit1
      d_composit1 i.inc* xsmk* xalc* i.exe* height weight* waist* hip* sbp* dbp* xglu* xchol* xhdl*
      xtri* xuric* xcre*)) hip* ///
(regress,                         include (i.sex  age* xedu*  xneu* xlymph* xplt* xhb* i.drnsaid*
      event_composit1 d_composit1 i.inc* xsmk* xalc* i.exe* i.drfat_sum* height weight* waist* hip* sbp*
      dbp* xglu* xchol* xhdl* xtri* xuric* xcre*)) sbp* ///
(regress,                           include (i.sex  age* xedu*  xneu* xlymph* xplt* xhb* i.drnsaid*
      event_composit1 d_composit1 i.inc* xsmk* xalc* i.exe* i.drfat_sum* height weight* waist* hip* sbp*
      dbp* xglu* xchol* xhdl* xtri* xuric* xcre*)) dbp* ///
(intreg,ll(zglu12_l)  ul(zglu12_u)          include (i.sex  age* xedu*  xneu* xlymph* xplt* xhb*
      event_composit1 d_composit1 i.inc* xsmk* xalc* i.exe* i.drfat_sum* weight* waist* hip*  sbp*  dbp*
      xglu* xchol* xhdl* xtri* xuric* xcre*)) xglu12 ///
(intreg,ll(zglu13_l)  ul(zglu13_u)          include (i.sex  age* xedu*  xneu* xlymph* xplt* xhb*
      event_composit1 d_composit1 i.inc* xsmk* xalc* i.exe* i.drfat_sum* weight* waist* hip*  sbp*  dbp*
      xglu* xchol* xhdl* xtri* xuric* xcre*)) xglu13 ///
(intreg,ll(zglu14_l)  ul(zglu14_u)          include (i.sex  age* xedu*  xneu* xlymph* xplt* xhb*
      event_composit1 d_composit1 i.inc* xsmk* xalc* i.exe* i.drfat_sum* weight* waist* hip*  sbp*  dbp*
      xglu* xchol* xhdl* xtri* xuric* xcre*)) xglu14 ///
(intreg,ll(zglu15_l)  ul(zglu15_u)          include (i.sex  age* xedu*  xneu* xlymph* xplt* xhb*
      event_composit1 d_composit1 i.inc* xsmk* xalc* i.exe* i.drfat_sum* weight* waist* hip*  sbp*  dbp*
      xglu* xchol* xhdl* xtri* xuric* xcre*)) xglu15 ///
(intreg,ll(zchol12_l)  ul(zchol12_u)    include (i.sex  age* xedu*  xneu* xlymph* xplt* event_composit1
      d_composit1 i.inc* xsmk* xalc* i.exe* i.drfat_sum* weight* waist* hip*  sbp*  dbp* xglu* xchol*
      xhdl* xtri* xuric* xcre*)) xchol12 ///
(intreg,ll(zchol13_l)  ul(zchol13_u)    include (i.sex  age* xedu*  xneu* xlymph* xplt* event_composit1
      d_composit1 i.inc* xsmk* xalc* i.exe* i.drfat_sum* weight* waist* hip*  sbp*  dbp* xglu* xchol*
      xhdl* xtri* xuric* xcre*)) xchol13 ///
(intreg,ll(zchol14_l)  ul(zchol14_u)    include (i.sex  age* xedu*  xneu* xlymph* xplt* event_composit1
      d_composit1 i.inc* xsmk* xalc* i.exe* i.drfat_sum* weight* waist* hip*  sbp*  dbp* xglu* xchol*
      xhdl* xtri* xuric* xcre*)) xchol14 ///
```

```
(intreg,ll(zchol15_l)  ul(zchol15_u)    include (i.sex  age* xedu*  xneu* xlymph* xplt* event_composit1
      d_composit1 i.inc* xsmk* xalc* i.exe* i.drfat_sum* weight* waist* hip*  sbp*  dbp* xglu* xchol*
      xhdl* xtri* xuric* xcre*)) xchol15 ///
(intreg,ll(zhdl12_l)  ul(zhdl12_u)          include (i.sex  age* xedu*  xneu* xlymph* xplt*
      event_composit1 d_composit1 i.inc* xsmk* xalc* i.exe* i.drfat_sum* weight* waist* hip*  sbp*  dbp*
      xglu* xchol* xhdl* xtri* xuric* xcre*)) xhdl12 ///
(intreg,ll(zhdl13_l)  ul(zhdl13_u)          include (i.sex  age* xedu*  xneu* xlymph* xplt*
      event_composit1 d_composit1 i.inc* xsmk* xalc* i.exe* i.drfat_sum* weight* waist* hip*  sbp*  dbp*
      xglu* xchol* xhdl* xtri* xuric* xcre*)) xhdl13 ///
(intreg,ll(zhdl14_l)  ul(zhdl14_u)          include (i.sex  age* xedu*  xneu* xlymph* xplt*
      event_composit1 d_composit1 i.inc* xsmk* xalc* i.exe* i.drfat_sum* weight* waist* hip*  sbp*  dbp*
      xglu* xchol* xhdl* xtri* xuric* xcre*)) xhdl14 ///
(intreg,ll(zhdl15_l)  ul(zhdl15_u)          include (i.sex  age* xedu*  xneu* xlymph* xplt*
      event_composit1 d_composit1 i.inc* xsmk* xalc* i.exe* i.drfat_sum* weight* waist* hip*  sbp*  dbp*
      xglu* xchol* xhdl* xtri* xuric* xcre*)) xhdl15 ///
(intreg,ll(ztri12_l)  ul(ztri12_u)          include (i.sex  age* xedu*  xneu* xlymph* xplt*
      event_composit1 d_composit1 i.inc* xsmk* xalc* i.exe* i.drfat_sum* weight* waist* hip*  sbp*  dbp*
      xglu* xchol* xhdl* xtri* xuric* xcre*)) xtri12 ///
(intreg,ll(ztri13_l)  ul(ztri13_u)          include (i.sex  age* xedu*  xneu* xlymph* xplt*
      event_composit1 d_composit1 i.inc* xsmk* xalc* i.exe* i.drfat_sum* weight* waist* hip*  sbp*  dbp*
      xglu* xchol* xhdl* xtri* xuric* xcre*)) xtri13 ///
(intreg,ll(ztri14_l)  ul(ztri14_u)          include (i.sex  age* xedu*  xneu* xlymph* xplt*
      event_composit1 d_composit1 i.inc* xsmk* xalc* i.exe* i.drfat_sum* weight* waist* hip*  sbp*  dbp*
      xglu* xchol* xhdl* xtri* xuric* xcre*)) xtri14 ///
(intreg,ll(ztri15_l)  ul(ztri15_u)          include (i.sex  age* xedu*  xneu* xlymph* xplt*
      event_composit1 d_composit1 i.inc* xsmk* xalc* i.exe* i.drfat_sum* weight* waist* hip*  sbp*  dbp*
      xglu* xchol* xhdl* xtri* xuric* xcre*)) xtri15 ///
(intreg,ll(zldl12_l)  ul(zldl12_u)          include (i.sex  age* xedu*  xneu* xlymph* xplt*
      event_composit1 d_composit1 i.inc* xsmk* xalc* i.exe* i.drfat_sum* weight* waist* hip*  sbp*  dbp*
      xglu* xtri* xldl* xuric* xcre*)) xldl12 ///
(intreg,ll(zldl13_l)  ul(zldl13_u)          include (i.sex  age* xedu*  xneu* xlymph* xplt*
      event_composit1 d_composit1 i.inc* xsmk* xalc* i.exe* i.drfat_sum* weight* waist* hip*  sbp*  dbp*
      xglu* xtri* xldl* xuric* xcre*)) xldl13 ///
(intreg,ll(zldl14_l)  ul(zldl14_u)          include (i.sex  age* xedu*  xneu* xlymph* xplt*
      event_composit1 d_composit1 i.inc* xsmk* xalc* i.exe* i.drfat_sum* weight* waist* hip*  sbp*  dbp*
      xglu* xtri* xldl* xuric* xcre* )) xldl14 ///
```

```
    (intreg,ll(zldl15_l)  ul(zldl15_u)          include (i.sex  age* xedu*  xneu* xlymph* xplt*
          event_composit1 d_composit1 i.inc* xsmk* xalc* i.exe* i.drfat_sum* weight* waist* hip*  sbp*  dbp*
          xglu* xtri* xldl* xuric* xcre*)) xldl15 ///
    (intreg,ll(zuric12_l)  ul(zuric12_u)    include (i.sex  age* xedu*  xneu* xlymph* xplt* event_composit1
          d_composit1 i.inc* xsmk* xalc* i.exe* weight* waist* hip*  sbp*  dbp* xglu* xtri* xldl* xuric*
          xcre*)) xuric12 ///
    (intreg,ll(zuric13_l)  ul(zuric13_u)    include (i.sex  age* xedu*  xneu* xlymph* xplt* event_composit1
          d_composit1 i.inc* xsmk* xalc* i.exe* weight* waist* hip*  sbp*  dbp* xglu* xtri* xldl* xuric*
          xcre*)) xuric13 ///
    (intreg,ll(zuric14_l)  ul(zuric14_u)    include (i.sex  age* xedu*  xneu* xlymph* xplt* event_composit1
          d_composit1 i.inc* xsmk* xalc* i.exe* weight* waist* hip*  sbp*  dbp* xglu* xtri* xldl* xuric*
          xcre*)) xuric14 ///
    (intreg,ll(zuric15_l)  ul(zuric15_u)    include (i.sex  age* xedu*  xneu* xlymph* xplt* event_composit1
          d_composit1 i.inc* xsmk* xalc* i.exe* weight* waist* hip*  sbp*  dbp* xglu* xtri* xldl* xuric*
          xcre*)) xuric15 ///
    (intreg,ll(zcre12_l)  ul(zcre12_u)      include (i.sex  age* xedu*  xneu* xlymph* xplt* xhb* i.drnsaid*
          event_composit1 d_composit1 i.inc* xsmk* xalc* i.exe* weight* waist* hip*  sbp*  dbp* xglu* xtri*
          xldl* xuric* xcre*)) xcre12 ///
    (intreg,ll(zcre13_l)  ul(zcre13_u)      include (i.sex  age* xedu*  xneu* xlymph* xplt* xhb* i.drnsaid*
          event_composit1 d_composit1 i.inc* xsmk* xalc* i.exe* weight* waist* hip*  sbp*  dbp* xglu* xtri*
          xldl* xuric* xcre*)) xcre13 ///
    (intreg,ll(zcre14_l)  ul(zcre14_u)      include (i.sex  age* xedu*  xneu* xlymph* xplt* xhb* i.drnsaid*
          event_composit1 d_composit1 i.inc* xsmk* xalc* i.exe* weight* waist* hip*  sbp*  dbp* xglu* xtri*
          xldl* xuric* xcre*)) xcre14 ///
    (intreg,ll(zcre15_l)  ul(zcre15_u)      include (i.sex  age* xedu*  xneu* xlymph* xplt* xhb* i.drnsaid*
          event_composit1 d_composit1 i.inc* xsmk* xalc* i.exe* weight* waist* hip*  sbp*  dbp* xglu* xtri*
          xldl* xuric* xcre*)) xcre15 ///
    (logit, iterate(100) augmen               include (i.sex  age* xedu*  xneu* xlymph* xplt*
          event_composit1 d_composit1 i.inc* weight* waist* sbp* dbp* i.drfat_sum* xglu* xchol* xhdl* xtri*
          )) drfat_sum* ///
,add(100)  noimpute force rseed(1234)
```

APPENDIX B

STATA COMMANDS FOR MULTIPLE MEDIATION ANALYSIS

- *Parallel multiple mediator model: Following* equations were constructed based on causal pathways:

  <u>Mediator models</u>

  - *Pathway A (see Supplementary Figure 1A)*

  Path a_1:

$$\ln\left[\frac{DM}{1 - DM}\right] = \beta_{01} + \beta_{11}NLR + \sum_i \delta_i Z_i$$

  Path a_2:

$$Cr = \beta_{02} + \beta_{12}NLR + \sum_i \delta_i Z_i$$

  Path a_3:

$$\ln\left[\frac{HT_1}{1 - HT_1}\right] = \beta_{03} + \beta_{13}NLR + \sum_i \delta_i Z_i$$

  Path a_4:

$$\ln\left[\frac{HT_2}{1 - HT_2}\right] = \beta_{04} + \beta_{14}NLR + \sum_i \delta_i Z_i$$

○ *Pathway B (see Supplementary Figure 1B)*

Path a_1:

$$\ln\left[\frac{DM}{1 - DM}\right] = \beta'_{01} + \beta'_{11}NLR + \sum_i \delta_i Z_i$$

Path a_2:

$$\ln\left[\frac{HT_1}{1 - HT_1}\right] = \beta'_{02} + \beta'_{12}NLR + \sum_i \delta_i Z_i$$

Path a_3:

$$\ln\left[\frac{HT_2}{1 - HT_2}\right] = \beta'_{03} + \beta'_{13}NLR + \sum_i \delta_i Z_i$$

Path a_4:

$$Cr = \beta'_{04} + \beta'_{14}NLR + \sum_i \delta_i Z_i$$

Outcome model

- *Pathway A (see Supplementary Figure 1A)*

Path a_1b_1 $\ln\left[\frac{\text{CVE}}{1 - \text{CVE}}\right] = \theta_{01} + \theta_{11}\text{NLR} + \theta_{21}\text{DM} + \sum_i \gamma_i Z_i$

Path a_2b_2 $\ln\left[\frac{\text{CVE}}{1 - \text{CVE}}\right] = \theta_{02} + \theta_{12}\text{NLR} + \theta_{22}\text{Cr} + \sum_i \gamma_i Z_i$

Path a_3b_3 $\ln\left[\frac{\text{CVE}}{1 - \text{CVE}}\right] = \theta_{03} + \theta_{13}\text{NLR} + \theta_{23}\text{HT}_1 + \sum_i \gamma_i Z_i$

Path a_4b_4 $\ln\left[\frac{\text{CVE}}{1 - \text{CVE}}\right] = \theta_{04} + \theta_{14}\text{NLR} + \theta_{24}\text{HT}_2 + \sum_i \gamma_i Z_i$

- *Pathway B (see Supplementary Figure 1B)*

Path a_1b_1 $\ln\left[\frac{\text{CVE}}{1 - \text{CVE}}\right] = \theta'_{01} + \theta'_{11}\text{NLR} + \theta'_{21}\text{DM} + \sum_i \gamma_i Z_i$

Path a_2b_2 $\ln\left[\frac{\text{CVE}}{1 - \text{CVE}}\right] = \theta'_{02} + \theta'_{12}\text{NLR} + \theta'_{22}\text{HT}_1 + \sum_i \gamma_i Z_i$

Path a_3b_3 $\ln\left[\frac{\text{CVE}}{1 - \text{CVE}}\right] = \theta'_{03} + \theta'_{13}\text{NLR} + \theta'_{23}\text{HT}_2 + \sum_i \gamma_i Z_i$

Path a_4b_4 $\ln\left[\frac{\text{CVE}}{1 - \text{CVE}}\right] = \theta'_{04} + \theta'_{14}\text{NLR} + \theta'_{24}\text{Cr} + \sum_i \gamma_i Z_i$

(HT_1: well-controlled HT; HT_2: poor-controlled HT)

- *Estimation of mediation effects*

The three mediator and outcome models were simultaneously combined using GSEM approach. Estimated coefficients were used to decompose the total effects of NLR on CVEs into mediated or IDE and DE (c') as follows:

The MEs comprised of

i) Mediated through DM (ME_{DM}) which could be calculated by summation of product coefficients of each pathway:

Pathway A: $a_1b_1 + a_1b_2b_8 + a_1b_3b_9 + a_1b_4b_5 + a_1b_4b_7b_9 + a_1b_4b_6b_8$

Pathway B: $a_1b_1 + a_1b_2b_9 + a_1b_3b_7 + a_1b_4b_8 + a_1b_3b_5b_9 + a_1b_4b_6b_9$

ii) Mediated through well-controlled HT (ME_{HT1}): The equation as follows

Pathway A: $a_3b_8 + a_1b_2b_8 + a_2b_6b_8 + a_1b_4b_6b_8$

Pathway B: $a_2b_7 + a_2b_5b_9 + a_1b_3b_7 + a_1 b_3b_5b_9$

iii) Mediated through poor-controlled HT (ME_{HT2}): The equation as follows

Pathway A: $a_4b_9 + a_1b_3b_9 + a_2b_7b_9 + a_1b_4b_7b_9$

Pathway B: $a_3b_8 + a_3b_6b_9 + a_1b_4b_8 + a_1 b_4b_6b_9$

iv) Mediated through Creatinine (ME_{Cr}): The equation as follows

Pathway A: $a_2b_5 + a_2b_6b_8 + a_2b_7b_9 + a_1b_4b_6b_8 + a_1b_4b_7b_9$

Pathway B: $a_4b_9 + a_1b_2b_9 + a_2b_5b_9 + a_3b_6b_9 + a_1b_3b_5b_9 + a_1b_4b_6b_9$

The total effects (TE) were estimated as $ME_{DM} + ME_{HT1} + ME_{HT2} + ME_{Cr} + c'$ and TE for each mediator as follows:

a) Total effect of NLR on CVEs through DM (TE_{DM})

Approach 1: $a_1b_1 + a_1b_2b_8 + a_1b_3b_9 + a_1b_4b_5 + a_1 b_4b_7b_9 + a_1b_4b_6b_8 + c'$

Approach 2: $a_1b_1 + a_1b_2b_9 + a_1b_3b_7 + a_1b_4b_8 + a_1b_3b_5b_9 + a_1b_4b_6b_9 + c'$

b) Total effect of NLR on CVEs through well-controlled HT (TE_{HT1})

Approach 1: $a_3b_8 + a_1b_2b_8 + a_2b_6b_8 + a_1b_4b_6b_8 + c'$

Approach 2: $a_2b_7 + a_2b_5b_9 + a_1b_3b_7 + a_1 b_3b_5b_9 + c'$

c) Total effect of NLR on CVEs through poor-controlled HT (TE_{HT2})

Approach 1: $a_4b_9 + a_1b_3b_9 + a_2b_7b_9 + a_1b_4b_7b_9 + c'$

Approach 2: $a_3b_8 + a_3b_6b_9 + a_1b_4b_8 + a_1 b_4b_6b_9 + c'$

d) Total effect of NLR on CVEs through Cr (TE_{Cr})

Approach 1: $a_2b_5 + a_2b_6b_8 + a_2b_7b_9 + a_1b_4b_6b_8 + a_1b_4b_7b_9 + c'$

Approach 2: $a_4b_9 + a_1b_2b_9 + a_2b_5b_9 + a_3b_6b_9 + a_1b_3b_5b_9 + a_1b_4b_6b_9 + c'$

The percent total MEs were estimated as follows:

i) Percent total ME or IDE of NLR through DM = $\frac{\mathrm{ME(DM)}}{TE}$ x 100

ii) Percent total ME or IDE of NLR through well-controlled HT = $\frac{\mathrm{ME(HT1)}}{\mathrm{TE}}$ x 100

iii) Percent total ME or IDE of NLR through poor-controlled HT= $\frac{\mathrm{ME(HT2)}}{\mathrm{TE}}$ x 100

iv) Percent total ME or IDE of NLR through Cr = $\frac{\mathrm{ME(Cr)}}{\mathrm{TE}}$ x 100

The percent of DE (% DE) = $c'/\mathrm{ME}_{\mathrm{DM}} + \mathrm{ME}_{\mathrm{HT}_1} + \mathrm{ME}_{\mathrm{HT}_2} + \mathrm{ME}_{\mathrm{Cr}} + c'$

The ORs of each mediation effect ($\mathrm{OR}_{\mathrm{ME}_{\mathrm{M}_i}}$) = $\exp^{\mathrm{ME}_{\mathrm{M}_i}}$

and 95% confidence interval (CI) of each mediation effect =

$$95\%\mathrm{CI}\ \mathrm{OR}_{\mathrm{ME}_{\mathrm{M}_i}} = \exp^{\left(\mathrm{ME}_{\mathrm{M}_i} \pm z_{\alpha/2}\sqrt{\mathrm{var}\left(\mathrm{ME}_{\mathrm{M}_i}\right)}\right)}$$

Generalized structural equation model (GSEM)

Pathway A

```
mi estimate, cmdok: gsem (outcomecve <- 1.htcont2 2.htcont2 nlr idmdef icre  drnsaid_use agegr ismk2 , family(bernoulli) link(logit))
        (htcont2 <-  nlr idmdef icre agegr   1.iedugr3 2.iedugr3 2.iinc 3.iinc  ialc2 drnsaid_use ///
        1.dlp2 2.dlp2 1.bmi3gr1 3.bmi3gr1 whrgr family(multinomial) link(logit)) ///
        (icre <- nlr idmdef  sex  iuric anemia, family(gau) link(iden)) ///
        (idmdef <- nlr 1.iedugr3 2.iedugr3  drnsaid_use 1.dlp2 2.dlp2 1.bmi3gr1 3.bmi3gr1 whrgr , family(bernoulli) link(logit)),
        vce(cluster empnm)
```

Pathway B

```
mi estimate, cmdok: gsem (outcomecve <- 1.htcont2 2.htcont2 nlr idmdef icre  drnsaid_use agegr ismk2 , family(bernoulli) link(logit))
        icre <- nlr 1.htcont2 2.htcont2 idmdef  sex iuric anemia, family(gau) link(iden)) ///
        (htcont2 <-  nlr idmdef  agegr  1.iedugr3 2.iedugr3 2.iinc 3.iinc ialc2 1.iexe 2.iexe ///
        drnsaid_use 1.dlp2 2.dlp2 1.bmi3gr1 3.bmi3gr1 whrgr iuric, ///family(multinomial) link(logit)) ///
        (idmdef <- nlr 1.iedugr3 2.iedugr3  drnsaid_use 1.dlp2 2.dlp2 1.bmi3gr1 3.bmi3gr1 ///
        whrgr , family(bernoulli) link(logit)), vce(cluster empnm)
```

Bootstrapping Commands for Pathway A

```
mi unset
gsem (outcomecve <- 1.htcont2 2.htcont2 nlr idmdef icre  drnsaid_use agegr ismk2 , family(bernoulli) link(logit))  ///
        (htcont2 <-  nlr idmdef icre agegr  1.iedugr3 2.iedugr3 2.iinc 3.iinc  ialc2 drnsaid_use 1.dlp2 2.dlp2 1.bmi3gr1 3.bmi3gr1 whrgr,
           family(multinomial) link(logit)) ///
        (icre <- nlr idmdef  sex  iuric anemia, family(gau) link(iden)) ///
        (idmdef <- nlr 1.iedugr3 2.iedugr3  drnsaid_use 1.dlp2 2.dlp2 1.bmi3gr1 3.bmi3gr1 whrgr , family(bernoulli) link(logit)),
        vce(cluster empnm)

gsem, coeflegend

ereturn list

mat list e(b)

program define nlrht, rclass

         tempname a1
         tempname a2
         tempname a3
         tempname a4
         tempname b1
         tempname b2
         tempname b3
         tempname b4
         tempname b5
         tempname b6
         tempname b7
         tempname b8
         tempname b9
         tempname c
```

```
mi estimate, cmdok: gsem (outcomecve <- 1.htcont2 2.htcont2 nlr idmdef icre  drnsaid_use agegr ismk2 , family(bernoulli)
                                      link(logit))  ///
                                 (htcont2 <-  nlr idmdef icre agegr   1.iedugr3 2.iedugr3 2.iinc 3.iinc  ialc2 drnsaid_use 1.dlp2 2.dlp2
                                      1.bmi3gr1 3.bmi3gr1 whrgr,  family(multinomial) link(logit)) ///
                                (icre <- nlr idmdef  sex  iuric anemia, family(gau) link(iden)) ///
                                (idmdef <- nlr 1.iedugr3 2.iedugr3  drnsaid_use 1.dlp2 2.dlp2 1.bmi3gr1 3.bmi3gr1 whrgr ,
                                      family(bernoulli) link(logit)), vce(cluster empnm)
  scalar `a1' = el(e(b_mi),1,77)
  scalar `a2' = el(e(b_mi),1,71)
  scalar `a3' = el(e(b_mi),1,31)
  scalar `a4' = el(e(b_mi),1,51)
  scalar `b1' = el(e(b_mi),1,5)
  scalar `b2' = el(e(b_mi),1,32)
  scalar `b3' = el(e(b_mi),1,52)
  scalar `b4' = el(e(b_mi),1,72)
  scalar `b5' = el(e(b_mi),1,6)
  scalar `b6' = el(e(b_mi),1,33)
  scalar `b7' = el(e(b_mi),1,53)
  scalar `b8' = el(e(b_mi),1,2)
  scalar `b9' = el(e(b_mi),1,3)
  scalar `c' = el(e(b_mi),1,4)
  return scalar total_dm =  abs((`a1'*`b1')+(`a1'*`b2'*`b8')+  ///
                                    (`a1'*`b3'*`b9') + (`a1'*`b4'*`b5')+ ///
                                    (`a1'*`b4'*`b6'*`b8')+(`a1'*`b4'*`b7'*`b9'))
  return scalar total_dm_1ht =  (`a1'*`b2'*`b8') + (`a1'*`b4'*`b6'*`b8')
  return scalar total_dm_2ht =  (`a1'*`b3'*`b9') + (`a1'*`b4'*`b7'*`b9')
  return scalar total_cr =  abs((`a2'*`b5')+(`a2'*`b6'*`b8')+  ///
                                   (`a2'*`b7'*`b9'))
  return scalar total_ht = abs((`a3'*`b8')+(`a4'*`b9'))
  return scalar total_1ht = abs((`a3'*`b8')+(`a1'*`b2'*`b8')+(`a1'*`b4'*`b6'*`b8')+ ///
                                   (`a2'*`b6'*`b8'))
  return scalar total_2ht = abs((`a4'*`b9')+(`a1'*`b3'*`b9')+(`a1'*`b4'*`b7'*`b9')+ ///
                                    (`a2'*`b7'*`b9'))
```

```
        return scalar direct_nlr =`c'
        return scalar total_effects = abs((`a1'*`b1')+(`a1'*`b2'*`b8')+  ///
                                  (`a1'*`b3' *`b9') + (`a1'*`b4' *`b5')+ ///
                                  (`a1'*`b4'*`b6'*`b8')+(`a1'*`b4'*`b7'*`b9')) + ///
                                  abs((`a2'*`b5')+(`a2'*`b6'*`b8')+(`a2'*`b7' *`b9')) + ///
                                  abs((`a3'*`b8')+(`a4'*`b9')) + abs(`c')
        return scalar or_direct_nlr = exp(`c')
        return scalar or_total_dm = exp((`a1'*`b1')+(`a1'*`b2'*`b8')+  ///
                                  (`a1'*`b3' *`b9') + (`a1'*`b4' *`b5')+ ///
                                  (`a1'*`b4'*`b6'*`b8')+(`a1'*`b4'*`b7'*`b9'))
        return scalar or_total_dm_1ht = exp((`a1'*`b2'*`b8') + (`a1'*`b4'*`b6'*`b8'))
        return scalar or_total_dm_2ht = exp((`a1'*`b3'*`b9') + (`a1'*`b4'*`b7'*`b9'))
        return scalar or_total_cr = exp((`a2'*`b5')+(`a2'*`b6'*`b8')+  ///
                                 (`a2'*`b7' *`b9'))
        return scalar or_total_ht = exp((`a3'*`b8')+(`a4'*`b9'))
        return scalar or_total_1ht = exp((`a3'*`b8')+(`a1'*`b2'*`b8')+(`a1'*`b4'*`b6'*`b8')+ ///
                                  (`a2'*`b6'*`b8'))
        return scalar or_total_2ht = exp((`a4'*`b9')+(`a1'*`b3'*`b9')+(`a1'*`b4'*`b7'*`b9')+ ///
                                  (`a2'*`b7'*`b9'))
end
nlrht
set seed 12345
bootstrap total_dm=r(total_dm) total_dm_1ht=r(total_dm_1ht) total_dm_2ht=r(total_dm_2ht) total_cr=r(total_cr) ///
          total_ht=r(total_ht) total_1ht=r(total_1ht) total_2ht=r(total_2ht)  ///
          direct_nlr=r(direct_nlr) total_effects=r(total_effects) direct_nlr2total=r(direct_nlr2total) ///
          total_dm2total=r(total_dm2total) total_cr2total=r(total_cr2total ) ///
          total_1ht2total=r(total_1ht2total) total_2ht2total=r(total_2ht2total) total_ht2total=r(total_ht2total) ///
          or_direct_nlr=r(or_direct_nlr) or_total_dm=r(or_total_dm) or_total_dm_1ht=r(or_total_dm_1ht) ///
          or_total_dm_2ht=r(or_total_dm_2ht) or_total_cr=r(or_total_cr)  ///
          or_total_ht=r(or_total_ht) or_total_1ht=r(or_total_1ht) or_total_2ht=r(or_total_2ht), reps(1000) cluster(empnm): nlrht

estat bootstrap, bc p norm
```
